# Potential socioeconomic impacts from ocean acidification and climate change effects on Atlantic Canadian fisheries

**DOI:** 10.1371/journal.pone.0226544

**Published:** 2020-01-10

**Authors:** Tyler J. B. Wilson, Sarah R. Cooley, Travis C. Tai, William W. L. Cheung, Peter H. Tyedmers

**Affiliations:** 1 School for Resource and Environmental Studies, Dalhousie University, Halifax, NS, Canada; 2 Ocean Conservancy, Washington, DC, United States of America; 3 Changing Ocean Research Unit, Institute for the Oceans and Fisheries, The University of British Columbia, Vancouver, B.C., Canada; Universidade de Vigo, SPAIN

## Abstract

Ocean acidification is an emerging consequence of anthropogenic carbon dioxide emissions. The full extent of the biological impacts are currently not entirely defined. However, it is expected that invertebrate species that rely on the mineral calcium carbonate will be directly affected. Despite the limited understanding of the full extent of potential impacts and responses there is a need to identify potential pathways for human societies to be affected by ocean acidification. Research on these social implications is a small but developing field. This research contributes to this field by using an impact assessment framework, informed by a biophysical model of future species distributions, to investigate potential impacts facing Atlantic Canadian society from potential changes in shellfish fisheries driven by ocean acidification and climate change. New Brunswick and Nova Scotia are expected to see declines in resource accessibility but are relatively socially insulated from these changes. Conversely, Prince Edward Island, along with Newfoundland and Labrador are more socially vulnerable to potential losses in fisheries, but are expected to experience relatively minor net changes in access.

## Introduction

Ocean acidification (OA) is a facet of climate change driven by increasing carbon dioxide (CO_2_) concentrations in the atmosphere and ocean [1,2]. Recognition of the potential for OA to act as a biological stressor in the marine environment has only been widely recognised in the past 15 to 20 years. Since then, research addressing its biological implications has expanded rapidly [2–4], suggesting the potential for wide-ranging effects on marine organisms and ecosystems. To date, findings have been highly variable with some species showing positive physiological responses while many others respond negatively; responses have also been seen to vary between populations of individual species [4,5]. Despite ranges of expected impacts, current understandings suggest that negative responses from marine species are likely to be more widespread than neutral or positive responses [3,4]. Furthermore, it is widely agreed that invertebrate species that rely on calcium carbonate shells and exoskeletons (e.g., corals, oysters, crabs) will likely express negative responses [3,4]. Low pH events, driven by upwelling and exacerbated by OA, have already impacted shellfish production in the Pacific Northwest of the United States and Canada [1–3,6–8].

Globally, seafood is a critical dietary component for over 3 billion people and is also a highly traded commodity [9,10];changes in production could therefore have significant impacts for many and diverse communities. There is a body of emerging literature seeking to address how OA may affect human systems by linking projected biological responses to OA with ecosystem services such as fisheries production. To date, socioeconomic OA impact research has been undertaken at a range of scales and resolutions from international [11–13] to national [14–16] and sub-national [17]. These studies have generally focused on OA in isolation from other climate change effects, and predicted declines in revenues and higher social risk in regions where shellfish fisheries are proportionally more important. Several previous assessments [e.g., 11,14,17], follow risk assessment frameworks to identify communities or regions that are most susceptible to impacts from OA. In these assessments risk is defined in conventions similar to that of the International Panel on Climate Change (IPCC) [18], and based on the socioeconomic structure of the social unit in question and the likelihood of an impact from OA.

**Fig 1:**
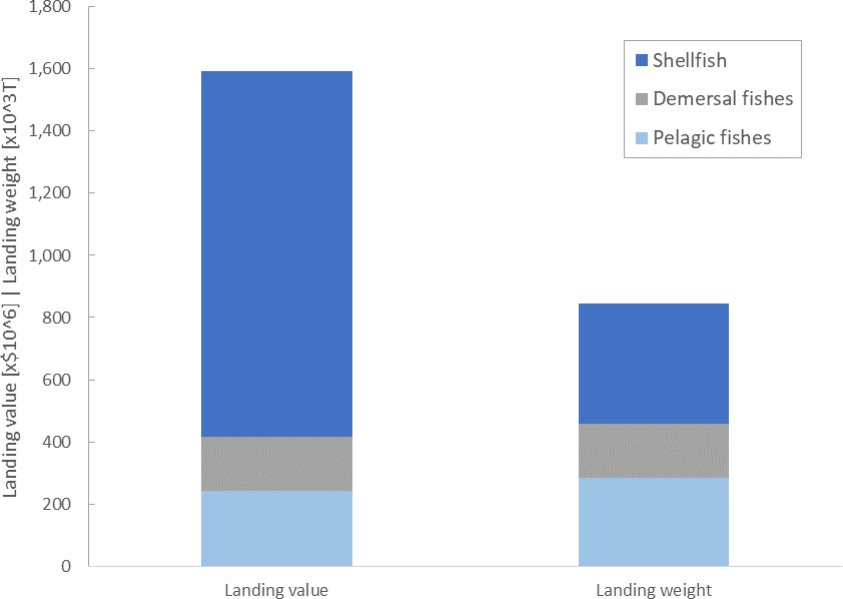
Average annual landings by species groups by dollar value and tonnage. Arranged by species group for Atlantic Canadian fisheries for 1991–2010. Dollar values are normalised to year 2000 dollars and are in millions of dollars, weights are presented in thousands of tonnes (both data types use the same axis). Includes production from aquaculture. Note, in these data ‘shellfish’ encompasses all harvested marine invertebrate species.

In Canada, seafood production is concentrated on the Atlantic Coast, with over 80% of total landings and over 85% of the commercial fishing fleet based in the region [19]. Within Atlantic Canada, shellfish have become an increasingly important component of capture fisheries, accounting for nearly 50% of regional landings by weight and over 75% of total landed value [20,21] (Fig 1). At the same time, Atlantic Canadian provinces and communities represent some of the least wealthy segments of the Canadian economy, with most of the provinces being net recipients of federal equalization payments as well as receiving overall above average per capita federal support [22]. Furthermore, most of the provinces in the region have comparatively rural populations, with many relatively small communities which are more highly dependent on employment from natural resource-linked sectors such as fisheries [23,24]. Given the high contribution of shellfish to total seafood production, together with the socioeconomic background of the provinces in the region, Atlantic Canada presents a highly relevant setting for investigating how potential OA-driven changes in fisheries might affect human communities.

In this paper, a biophysical model was linked with an impact assessment framework to investigate how OA and climate change might drive shifts in future availability of fisheries resources for Atlantic Canada, and which provinces in the region are most likely to be affected by these shifts. The assessment aims to highlight regions in Atlantic Canada where potential impacts from OA and climate change is highest, under contemporary socioeconomic settings, given predicted changes in fisheries’ access. In the assessment framework OA and climate change are the sole drivers for changes in fisheries. Given the limits of scientific understanding and modelling capabilities more subtle, albeit important, factors such as potential for individual species’ evolutionary responses to changing environmental conditions are not included.

## Methods

### Study area

**Fig 2:**
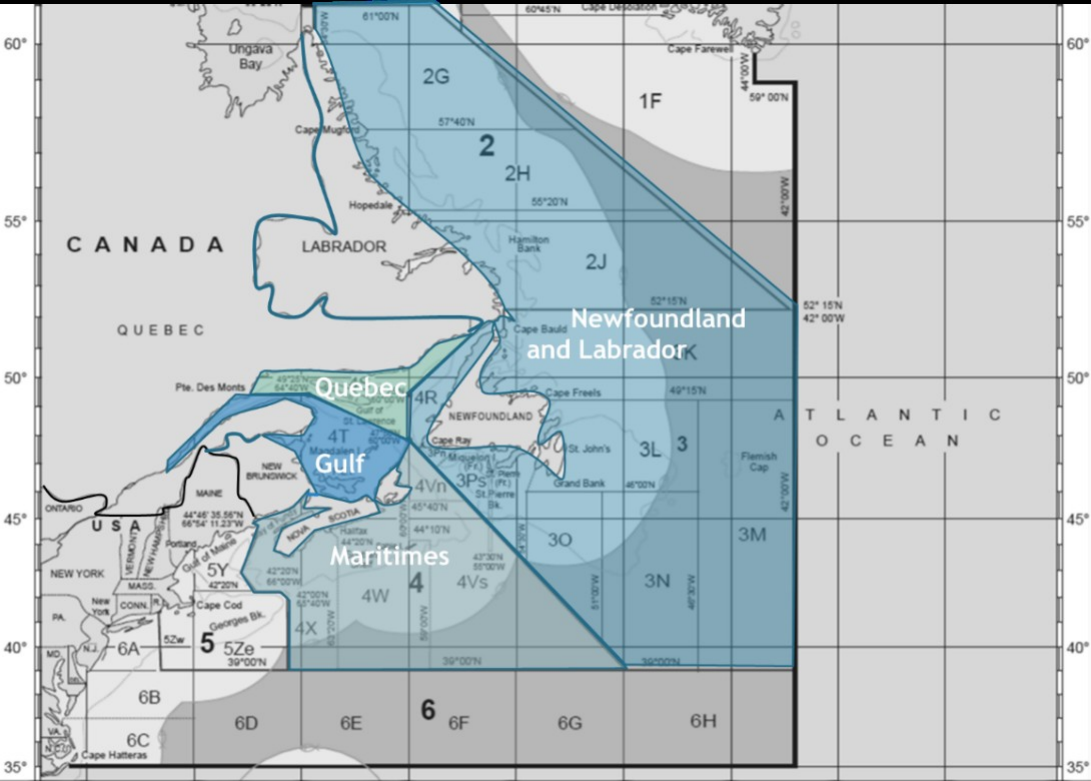
Eastern Canada as it relates to the Northwest Atlantic Fisheries Organization (NAFO) fishing areas with DFO management areas overlaid. For clarity approximate provincial borders have been emphasised in dark blue, U.S. and Canada border has been emphasised in black. In this assessment Gulf and Quebec management areas were treated as a single unit (figure modified from [25]).

‘Atlantic Canada’ typically refers to four Canadian provinces: New Brunswick (NB), Newfoundland and Labrador (NL), Nova Scotia (NS) and Prince Edward Island (PEI). The province of Quebec (Que) also borders the Atlantic Ocean but it is often not included as part of ‘Atlantic Canada’ due to significant demographic and cultural differences. However, for the purposes of this research, ‘Atlantic Canada’ will also include Quebec, as the province operates fisheries in Atlantic waters and is therefore exposed to potential changes in fisheries (Fig 2).

All Canadian marine waters fall under federal jurisdiction and are managed by Fisheries and Oceans Canada (DFO). In Atlantic Canada, marine waters are divided into four management areas: Newfoundland and Labrador, Maritime, Gulf, and Quebec (Fig 2). In this analysis the Gulf and the Quebec management areas are treated as a single unit (henceforth, collectively referred to as the Gulf management area) because both management areas are comparatively small and the biophysical model used to project distribution changes does not have sufficient resolution to reliably differentiate between these management areas. The provinces of NS and NB border two management areas (Maritime and Gulf; Fig 2), and landings for each area are reported separately.

### Species selection

Fisheries in Atlantic Canada target a diverse array of marine species. Due to the variable nature of anticipated responses to OA a subset of commercially harvested species was selected to assess the potential impacts on fisheries and, ultimately, Atlantic Canadian communities. The selection of species was based on a combination of: a) their current economic importance (including capture fisheries and aquaculture production) in Atlantic Canada; and b) the current understanding that shellfish species are more susceptible to OA than finfish species [3,4].

To guide the selection of the specific fisheries that would be the focus of this research, total annual landings for all fisheries in each province and management area were compiled. Fisheries landing weights and values data were collected from DFO statistics [20]. Annual landing weights from 1991–2010 were averaged to obtain baseline annual weight for the year 2000. Landing values for each year were normalised to year 2000 dollars using the consumer price index from the Bank of Canada [26], and similarly averaged across 1991–2010.

The compiled landing values included aquaculture production. While the biophysical model projections are not directly applicable to aquaculture, the production values were included here for two reasons. First, the majority of shellfish aquaculture in the region relies on wild populations for recruitment [27]. Consequently, changes that affect wild population distributions will also potentially affect levels of aquaculture production. And second, DFO data for American oyster merges aquaculture and wild harvest production statistics. Moreover, PEI blue mussel data reporting changed from being reported as capture to being reported as aquaculture midway through the baseline time period.

Aquaculture production data is available at a provincial level but, in contrast to wild harvest, it is not differentiated between management areas. Therefore, the data for NS shellfish aquaculture production values were evenly divided between the Gulf and Maritime management areas because aquaculture production in NS was distributed across the whole province. For NB, shellfish aquaculture was all counted under the Gulf management area because NB shellfish aquaculture is concentrated in that management area–in contrast to NB finfish aquaculture, which occurs in the Bay of Fundy (i.e., the Maritime management area).

### Biophysical impact modeling

#### Model selection

To highlight potential impacts of OA on the fishery sector, shellfish fisheries were prioritised for assessment in this research. However, other aspects of climate change will also drive shifts in species abundances and distributions and will add to and potentially interact with OA effects [28–30]. Therefore, it was decided that inclusion of climate change related impacts along with OA would present a more holistic estimation of the potential future of Atlantic Canadian shellfish fisheries. A Dynamic Bioclimate Envelope Model (DBEM) [31,32], which integrates species’ ecophysiology and biogeographical responses to changes in ocean conditions (including OA, warming and deoxygenation) to predict future species distributions, was selected to inform the underlying biophysical conditions driving potential changes in landings in Atlantic Canadian fisheries.

#### Model data

The DBEM used in this study was initially developed in 2008 [32] and subsequently modified and updated [31,33,34]. The DBEM uses outputs from earth system models as environmental drivers to project spatial and temporal changes in species distribution, abundance, and catch potential. It integrates the oxygen- and capacity-limited thermal tolerance [35] and the gill-oxygen supply [36] hypotheses to estimate the impacts of environmental stressors—including OA—on somatic growth and mortality rates. The model outputs used here incorporated OA as an impact on growth and survival for mollusc and crustacean species. Specifically, mollusc and crustacean species groups had different impact levels per unit change in pH based on the mean impact findings of meta-analyses conducted in 2010 and 2013 [3,4] [31,34,37]. Potential for evolutionary responses to changing conditions are not accounted for in the current iteration of the DBEM. For additional details on the DBEM see S1 Note.

Outputs from the model were structured as annual species-specific maximum catch potentials (a proxy for maximum sustainable yield) for cells on a global half-degree latitude by half-degree longitude grid for the period of 1950 to 2100. For each species and year combination, there were 12 datasets generated by the model representing: two Radiative Concentration Pathways (RCP) scenarios for future CO_2_ emissions [38,39] (RCP 2.6 –‘highly mitigated CO_2_ emissions’ and RCP 8.5 –‘business as usual CO_2_ emissions’); two OA treatments (‘with OA’; and ‘without OA’); and using three separate climate models (NOAA’s Geophysical Fluid Dynamics Laboratory (GFDL-ESM), Institute Pierre Simon Laplace Climate Modelling Centre (IPSL-ESM) and Max Planck Institute for Meteorology (MPI-ESM)) (Table 1).

**Table 1:** Schematic representation of data configuration for each species, *s*. Each of the 12 configurations yielded a separate global distribution of catch potential in half-degree latitude by half-degree longitude cells for each year, *Y* that was modelled (1950–2100).

Species ***s***, Year ***Y***	RCP 2.6	with OA	GFDL-ESM
			IPSL-ESM
			MPI-ESM
		without OA	GFDL-ESM
			IPSL-ESM
			MPI-ESM
	RCP 8.5	with OA	GFDL-ESM
			IPSL-ESM
			MPI-ESM
		without OA	GFDL-ESM
			IPSL-ESM
			MPI-ESM

Data extraction for the species of interest along with the following data manipulation was performed using Mathworks MATLAB, version R2015b, and Microsoft Excel 2013. Initially, the 12 global datasets for each of the selected species were geographically constrained to include only data from grid cells corresponding to Canada’s Atlantic EEZ (NAFO areas 2, 3 and 4; Fig 2). Modifying from earlier methods, 20 year running means were calculated for each decade (e.g., for the year 2000, values were averaged from 1991–2010) for each species’ 12 geographically truncated datasets in order to smooth interannual variability from the climate model projections [40]. This was performed for each grid cell in the truncated datasets.

**Fig 3:**
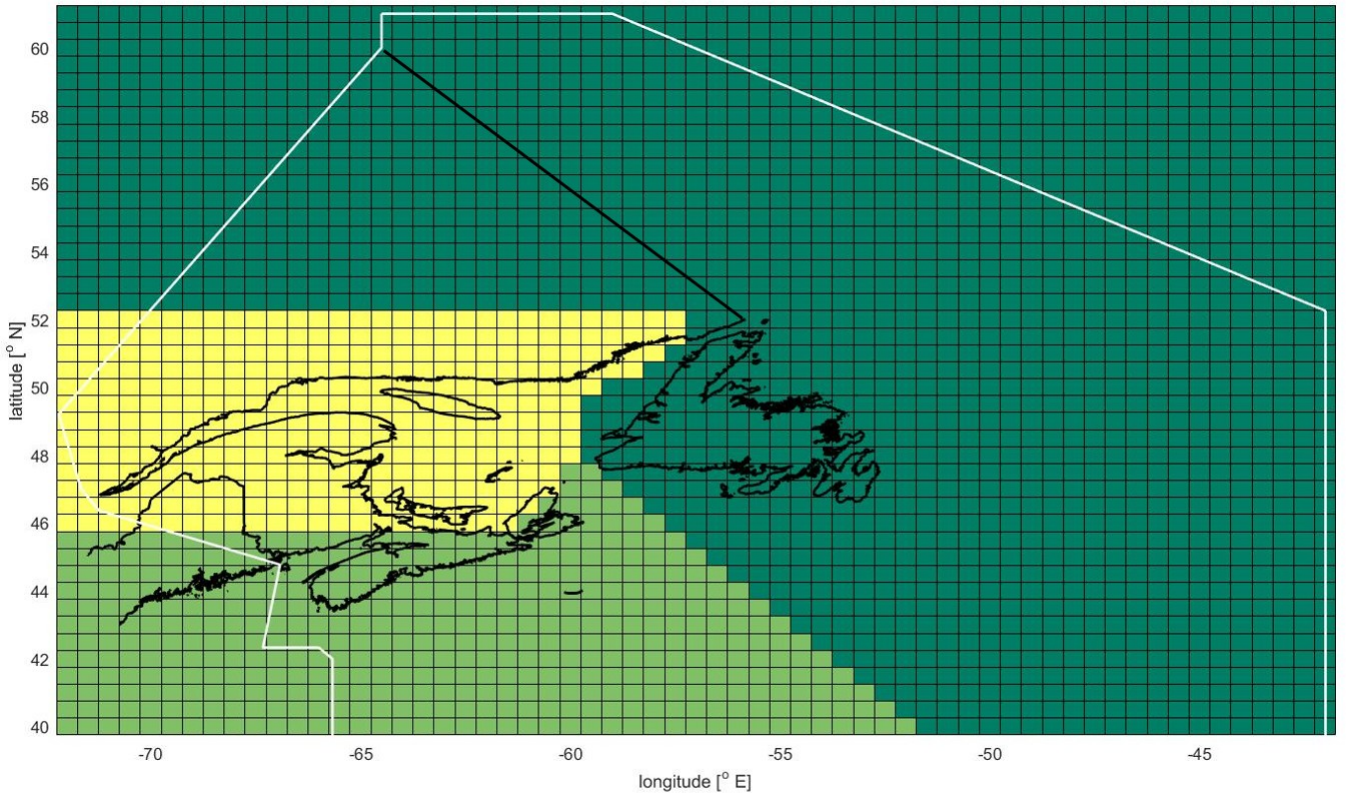
Geographic coverage of datasets. Grid lines indicate individual half-degree latitude/longitude data cells. White boundary line outlines study area (i.e., Canadian Atlantic EEZ). Data outside of this boundary was truncated from the datasets before aggregating management area data. Cell colours define data aggregation layer for DBEM outputs: Newfoundland and Labrador management area is represented by dark green cells; Maritime management area is represented by light green cells, and Gulf (including Quebec) management area is represented by the yellow-green cells. Inclusion of land area within data aggregation is irrelevant, as these cells do not contain any data. Coastline data from: [41].

Median values from outputs using each of the three earth system models were used to limit the uncertainty surrounding model variability (the following steps were also performed for the datasets generated from the individual climate models, these are available in the Supporting Information S1 Table). This data manipulation resulted in a total of four datasets (two RCP scenarios and two OA treatments) for each species. The catch potentials were then summed across the DFO management areas (as per Fig 3) for each decade. Minor exceptions to this process were necessary where the median dataset projected future abundance of a given species in a management area dropping to zero. In these instances, the mean of the management area aggregated data from the three climate model values was substituted.

To project future landings, DFO reported landings for the baseline period were multiplied by the relative change indicated by the outputs of the DBEM. The year 2000 was used as the reference year against which future changes in potential landings were assessed. Relative change in the modelled catch potential for each species in subsequent decades was calculated for each management area, as well as for the entire Atlantic Canadian region (Eq 1). To assess patterns across the region, the relative change for each grid cell was also calculated.

$$\Delta_{A,Y,r,s}=\frac{Y_{data}-2000_{data}}{2000_{data}}$$ Eq 1

Where *Δ*_*A*,*Y*,*s*_ is the relative change compared to the year 2000 for management area *A*, in decade *Y*, under RCP scenario r, and species *s*.

#### Analysis of potential future landings

Two future time-steps, representing the middle and end of the 21^st^ century (2050 and 2090, respectively) were selected as endpoints for the assessment of changes in landings. Values for both time-steps were calculated relative to the reference year (2000).

The coupled impact of climate change including OA was determined to be a more relevant focus for the investigation of potential impacts across Atlantic Canada and was therefore used for the main analysis. The 2050 and 2090 potential landings for each species within each management area were compared with baseline landings. Differences in future landings under the two climate scenarios (RCP 2.6 and RCP 8.5) were also compared to identify how different emission scenarios might affect future fisheries. Individual species changes, as well as cumulative changes across species, were investigated within and between the management areas.

### Constructing the socioeconomic impact index

An impact assessment framework was developed to evaluate the potential socioeconomic impacts Atlantic Canada may experience through changing fisheries landings driven by ocean acidification and climate change. A range of approaches to modelling potential socioeconomic impacts posed by hazards such as OA and climate change have emerged in the literature in recent years [14,17,18]. The framework constructed here broadly follows the methodology of risk assessments addressing ocean acidification’s impacts on fisheries [17], wherein risk is defined as the intersection of a hazard, exposure to the hazard, and vulnerability to the hazard (following the IPCC Working Group II report: Managing the Risks of Extreme Events and Disasters to Advance Climate Change Adaptation—Chapter 2: Determinants of Risk: Exposure and Vulnerability. [18]). Vulnerability in turn encompassed two sub-components: sensitivity and adaptive capacity. This assessment follows similar definitions with the potential impact being defined as the combination of social vulnerability and biophysical exposure to the hazard of climate change and ocean acidification. The potential impact is not an absolute measurement, rather it is a metric for a relative comparison of how susceptible the provinces are to changing conditions.

**Fig 4:**
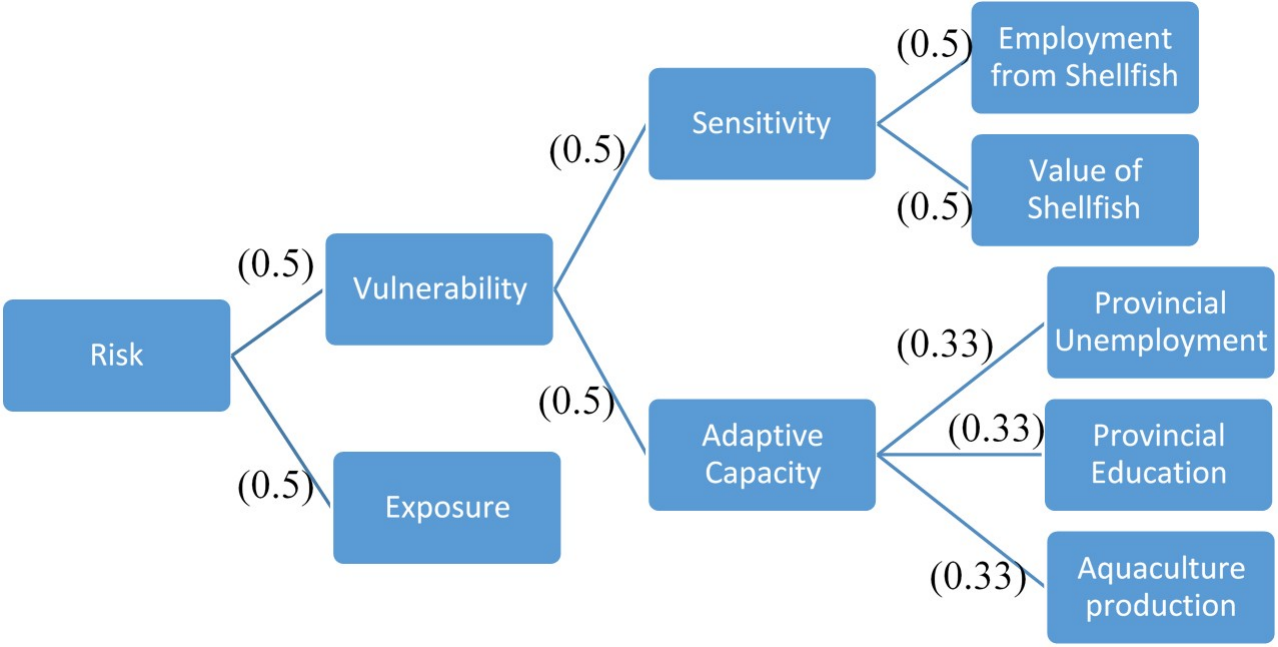
Framework for the impact index demonstrating relationship between components. Elements at each branch are equally weighted to other items at the same level as indicated by bracketed numbers.

In the framework developed for Atlantic Canada, the exposure component represented the modelled OA and climate change-driven effects on the fisheries as determined through analysis of DBEM outputs. The sensitivity and adaptive capacity terms were used to describe the social factors that may obstruct or fortify communities’ responses to changes in fisheries. In total, the impact index was informed by six separate indicators: one for exposure, two for sensitivity, and three for adaptive capacity (Fig 4). Within each individual component of the framework, the sub-components or indicators were equally weighted. This weighting inherently implies that the indicators contribute equally to potential impacts in the region. In reality, this is unlikely to be the case, however without strong support for preferential weights, equal weighting was selected as the most straightforward and neutral system [42,43] (Fig 4).

Although a cumulative impact index was constructed, the biophysical and social factors should be considered as representing two distinct aspects of potential impact [14,18]. The combined index represents where these two dimensions overlap to have the highest effect. The biophysical indicator (i.e., exposure) represents where the most prominent changes in the resource will occur; and the social indicators (i.e., vulnerability) highlight where the expected changes are likely to have the highest impact given the current socioeconomic setting.

Two of the provinces, NB and NS, border two separate DFO management areas (the Gulf and the Maritime management areas; Fig 2). For the purposes of this analysis, these two provinces were sub-divided into gulf (NB-gulf; NS-gulf) and maritime (NB-mar; NS-mar) provincial sub-sections. However, both sub-sections of the respective provinces rely on the same social data because these data were not available at a resolution that would have allowed separate treatments within the provinces. The sub-sections were exposed separately to potential changes in both relevant DFO management areas.

#### Exposure indicator construction

The most significant divergence from previous risk/vulnerability assessments of potential impacts from OA was the inclusion of a biophysical model to inform the exposure and hazard components of the framework simultaneously. Past assessments have used an expected change in ocean chemistry to represent OA (the hazard) and used this in concert with the importance of OA susceptible fisheries (the exposure) to link the OA phenomenon with fisheries [11,14,15,17]. In this assessment, the biophysical model directly applied the expected climate change impact (including OA) onto the relevant fisheries. This allowed the change in fisheries landings to be explicitly incorporated into the framework. Thus the hazard and exposure terms were essentially incorporated into a single term (collectively referred to as ‘exposure’ throughout this assessment).

A single indicator to account for all of the assessed species was constructed. To reflect the very different current economic contributions of individual species to total landed value, the proportion of the total shellfish value derived from each species was calculated and multiplied by its DBEM-predicted relative change for each management area. These value-scaled changes were summed across all assessed species (Eq 2). This was repeated separately for 2050 and 2090 and both RCP climate scenarios to obtain four exposure indicator scores per management area. From here forward, the term ‘quartet’ will be used to refer to the four exposure scenarios, as well as the subsequent potential impact scores.

$$E_{A,Y,r}={\sum \left[\Delta_{A,Y,r,s}\times \left(\frac{V_{A,s}}{V_{A,tot}}\right)\right]}_{S}$$ Eq 2

Where *E*_*A*,*Y*,*r*_ is the exposure indicator score for area *A*, in time-step *Y*, and RCP scenario *r*. *Δ*_*A*,*Y*,*s*_ is the DBEM predicted relative change in landings for time-step *Y*, RCP scenarios *r*, and species *s (*from Eq 1*)*. *V*_*A*,*s*_ is the year 2000 average annual value for species *s*, and *V*_*A*,*tot*_ is the total annual landings of the seven species value for the management area (Table 2).

**Table 2:** Cumulative exposure indicator of future changes in fisheries (before normalisation of values). Values in fourth and fifth columns are the indicator scores (i.e., the relative change in landings scaled by proportional value of the individual species). For integration into the impact index, the exposure scores were reversed and then normalised to score between 0 and 1, so that declines in landings earned a high score and implied a high exposure to OA and climate change.

Management region	Exposed provinces	Climate scenario	Exposure indicator scores	
			2050	2090
Maritime	NS-mar; NB-mar	RCP 2.6	-0.114	-0.166
		RCP 8.5	-0.202	-0.498
Gulf	NS-gulf; NB-gulf; PEI; Que	RCP 2.6	0.042	0.017
		RCP 8.5	0.070	0.033
Newfoundland and Labrador	NL	RCP 2.6	-0.012	-0.026
		RCP 8.5	0.015	0.060
Regional total	n/a	RCP 2.6	-0.014	-0.034
		RCP 8.5	-0.018	-0.069

The impact index was constructed so that high scores implied higher potential for impact. To align the exposure scores with this orientation, the values were multiplied by (-1) so that losses in potential future landings became positive. Additionally, in order to combine the separate index indicators, it was necessary to first normalise each separate indicator to score between 0 and 1 [42,43]. Therefore, the 12 exposure values (three management areas, each with a quartet of scores) were normalised following a min-max calculation to score between 0 and 1 (Eq 3) [43]. Min-max normalization is a widely used method to allow distinct indicators with different units to have the same range of values (i.e., 0–1) for more straightforward integration into an index [43]. In the index, the exposure indicator scores for management areas were combined with the social vulnerability indicator scores for adjacent provinces–the Gulf and the Maritime management areas’ scores were applied to more than one province (NB-gulf, NS-gulf, PEI, and Que; and NB-mar, NS-mar; respectively) while the Newfoundland and Labrador management area scores were only applied to NL (Fig 3).

$$I_{E,A,Y,r}=\frac{\left(E_{A,Y,r}-\text{Min}(E)\right)}{\left(\text{Max}(E)-\text{Min}(E)\right)}$$ Eq 3

Min-Max approach for normalising exposure indicator scores [43]. Where *I*_*E*,*A*,*Y*,*r*_ is the normalised index score for the exposure score *E*_*A*,*Y*,*r*_ as calculated in Eq 2. Min*(E)* and Max*(E)* indicate the respective minimum and maximum exposure scores obtained. The normalisation sets the maximum score to be 1 and the minimum score to be 0.

#### Social vulnerability

Vulnerability represents a province’s reliance on fisheries (sensitivity) as well as the community’s broader ability to respond to, and absorb, changes (adaptive capacity). Vulnerability was assessed at the provincial level as this was the finest level of political organization for which most relevant data were consistently available across the region. To align the social data with the exposure data, the year 2000 was used as the baseline where possible. However, some of the data (e.g., crew size) was drawn from reports which are not published annually, and consequently these data are from the nearest year available (in all instances this was within 10 years of the year 2000).

The social data was centered on the year 2000 baseline to align with the biophysical data. Potential changes in the socioeconomic structures for the region were not considered. While it is recognised that in reality social systems will certainly respond and adapt in response to changing resource availability. The intent of this assessment was to establish the relative importance of the resources in the baseline year in order to identify which provinces are more likely to be impacted by, and require responses to, changes in resources.

***Sensitivity*** of each province was based on the importance of the assessed fisheries to provincial economies and social structures. Compared to other Canadian provinces, fisheries in Atlantic Canada represent a much more important economic sector as well as a source of livelihood in rural communities. Each province was scored on two indicators: a) the economic value derived from the seven species relative to total provincial GDP, and b) the employment directly associated with harvesting the selected species (Fig 4). Given the particularly high value of some fisheries in the region (e.g., lobster) the economic value of the fisheries provides insight on the relative importance of the industry to the provinces. Whereas the amount of employment that is tied to fisheries better reflects the sensitivity of communities. In reality these two indicators may not be entirely independent from each other, however given the provincial economies and the distinct management structures of the separate fisheries (e.g., two large vessels harvest nearly all of the surf clam in NS-mar, whereas the lobster fishery is dominated by far more numerous but smaller vessels with fewer than five crew members) they were treated as representing these two distinct aspects of the provincial socioeconomic structure.

The first indicator was assessed using year 2000 total annual landed value (averaged from 1991–2010) of the assessed species as a fraction of total provincial GDP (averaged between 1997 and 2003). To make the dollar values comparable across years all dollar values were normalised to year 2000 dollars [26,44]. Scaling the fisheries value relative to provincial GDP allowed the indicator scores to be relatable between the provinces, which have a broad range of economic scales.

It is difficult to obtain realistic employment estimates for individual fisheries because of the seasonal nature of the sector. This type of data is further convoluted by different methods of reporting employment statistics. For example, employment data are often presented as full-time equivalent jobs (a measure of the number of hours of work) in government reports. This is not a particularly relevant metric for seasonal employment–especially when it can generate enough income that additional employment is not necessary to support a desired quality of life (as can be the case for high-value fisheries). The estimates used here attempt to reflect the number of people employed with fisheries as their main source of income.

An estimate of employment was derived from the number of licences per species multiplied by average crew sizes for the applicable fishing fleet. Licence numbers were obtained from DFO statistics [45]. Where possible crew size estimates were collected from industry reports and assessments [46–50] (see S2 Note for an aside on ‘inshore’ and ‘offshore’ fleets and special cases regarding data availability). As well as through discussion with Michael Gardner, of Gardner Pinfold Consulting (personal communication, September 11, 2017). Crew size estimates for most of the assessed fisheries (in their respective management areas) were obtained from studies conducted in the early 2000s but these were the closest available data to the baseline year (see S2 Table for data sources for crew size estimates). Where crew size estimates were not available for a given species within a specific management area, the average crew size from fisheries for that species from other areas was used. Total employment in the relevant fisheries was summed for each province and divided by provincial population.

Aquaculture employment was not considered in this indicator because available data does not readily differentiate between finfish and shellfish aquaculture employment. Furthermore, employment from aquaculture is relatively small compared to wild harvest employment. As a result employment dependence was set to zero for species whose production was derived entirely or almost entirely from aquaculture. For similar reasons, estimates of secondary employment such as processing and retail were not considered in this assessment.

For many fisheries in the Atlantic region licences are not fully utilised. However, given the high value of the species assessed in this study, it was considered reasonable to assume all or a very high proportion of all licences in these fisheries were active. Crossover between fisheries, where a licence holder (as well as crew) may operate in more than one of the assessed fisheries was not accounted for (i.e., double counting was a possibility). Lacking a much more thorough social investigation this indicator provides an approximation of the employment derived from shellfish harvesting in the region. Assuming employment patterns in the industry are similar between provinces, using primary harvesting as a proxy for total employment should provide a reasonable first-order estimate for comparing relative importance of the fisheries between provinces.

The values obtained for both sensitivity indicators were normalised (min-max) to score between 0 and 1, with the province where the fisheries are proportionally most important scoring 1 for each indicator. For each province the two indicator scores were averaged (Fig 4) to obtain a final sensitivity score with a maximum possible score of 1 (Eq 4). A high score indicated a higher reliance on the fisheries, and a more sensitive social unit.

$$Sen_{P}=\frac{\left(Emp_{NP}+Val_{NP}\right)}{2}$$ Eq 4

Where *Sen* is the sensitivity score for province *P*, and *Emp*_*NP*_ is the normalised employment indicator and *Val*_*NP*_ is the normalised economic value indicator for province, *P*.

***Adaptive capacity***: To estimate the ability of each province to potentially respond to changes in fisheries landings, three indicators of adaptive capacity were compiled. Adaptive capacity is typically representative of positive aspects of a society [18], so the indicators were collected with higher scores indicating greater adaptive capacity. The indicator values were then reversed so that a lower adaptive capacity contributed to a higher vulnerability.

In other socioeconomic assessments of potential OA effects on fisheries, an indicator representing alternative employment options has been used as a key component of adaptive capacity [14,17]. This is similarly relevant for fisheries in Atlantic Canada, if the availability of fisheries resources decline people currently employed in the industry will need to shift to other employment sectors. As previously mentioned, due to the seasonal nature of fisheries employment in Atlantic Canada it is difficult to distinguish patterns of employment in fisheries as separate from employment in other sectors. Thus, provincial unemployment rates were used to indicate the potential for alternative employment options to fishing [15,17] following an assumption that low provincial unemployment would mean a higher demand for potentially displaced fishers. Provincial unemployment is particularly relevant in Atlantic Canada where rates were 3% to 10% above national rates during the assessment baseline year [51]. Data were averaged across a 10 year period bracketing the year 2000 baseline for the assessment (i.e. 1996–2005 - [51]). The reciprocal of provincial unemployment values were used so that lower provincial unemployment increased adaptive capacity.

Education within a society provides an indication of how well a community will be able to respond to changing conditions and has been used in other socioeconomic assessments of OA [15,17]. Higher education levels are generally seen as presenting more opportunities to adapt, while lower education rates limit options and capabilities to respond to change. The percent of adult populations with at least some post-secondary education was used to estimate the education level of the five assessed provinces [52]. As with the unemployment data, provincial education were averaged from 1996 to 2005.

The final element that was considered as part of each province’s adaptive capacity was the extent to which potentially OA-impacted species are currently cultured in the region. Aquaculture production is expected to be more resilient to OA and climate change than wild harvest, since many environmental conditions can be at least partially controlled or compensated for–especially when hatcheries are used to rear organisms through the most susceptible life-stages [53]. Thus, a strong aquaculture sector could potentially strengthen a community’s ability to respond to OA and climate change.

In Atlantic Canada there is no aquaculture production of crustacean species so the aquaculture indicator only includes mollusc production. The indicator was constructed to reflect patterns in development within the industry as well as relative contribution to total provincial shellfish production. First, the trend in mollusc aquaculture production was estimated for each province by comparing the average annual production for 2001 to 2010 against average annual production for 1991–2000. Second, the fraction of total shellfish production (including both wild harvest and aquaculture) sourced from mollusc species (using the year 2000 average annual production) was calculated. These two terms were then multiplied together to obtain the indicator value (Eq 5).

$$Aq_{p}=\sqrt{\left(\frac{Aqua_{2001-2010}}{Aqua_{1991-2000}}\right)\times \left(\frac{Mol_{1991-2010}}{Tot_{1991-2010}}\right)}$$ Eq 5

Where *Aq*_*p*_ is the aquaculture indicator score for province *p*. *Aqua* denotes the average aquaculture production for the subscripted time-period; *Mol* indicates mollusc production; and *Tot* indicates total shellfish production.

As with the previous index components, each of the adaptive capacity indicators were transformed linearly to score between 0 and 1. To align with the directionality of the other components in the framework the normalised scores were subtracted from 1 so that weaker adaptive capacity indicators had higher scores (i.e., closer to 1) and therefore contributed to the potential impact. As with the indicators for sensitivity, the individual adaptive capacity indicators were treated as having equal weights. The three adaptive capacity indicators (for employment, education and aquaculture production) were averaged to obtain a cumulative adaptive capacity score with a maximum potential value of 1 Eq 6.

$$AC_{P}=\frac{\left(UE_{NP}+Ed_{NP}+Aq_{NP}\right)}{2}$$ Eq 6

Where *AC* is the adaptive capacity score for province P, and *UE*_*NP*_ is the normalised provincial unemployment indicator, *Ed*_*NP*_ is the normalised education level indicator and *Aq*_*NP*_ is the normalised aquaculture indicator.

**Vulnerability**: Sensitivity and adaptive capacity were treated as having equal weights, and their values were averaged to arrive at a social vulnerability score for each province, with a potential maximum value of 1 (Fig 4). Weighting the sensitivity and adaptive capacity components equal to each other indirectly applied different weights to their composite indicators (Fig 4). However, the concepts of sensitivity and adaptive capacity represent different aspects of social vulnerability so their overall contributions were treated as equally relevant regardless of the number of indicators.

$$Vuln_{p}=\frac{\left(Sen_{P}+AC_{P}\right)}{2}$$ Eq 7

Where *Vuln*_*P*_ is the vulnerability score, *Sen*_*P*_ is sensitivity (Eq 4) and *AC*_*P*_ is adaptive capacity (Eq 6) for province *P*.

#### Potential socioeconomic impact

To calculate the final impact index scores the exposure and vulnerability scores were equally weighted and then combined. Each province (or sub-section thereof for NB and NS) received a quartet of potential impact scores following the quartet of exposure indicator scores that were applied. The 28 final index scores were divided into five categories of potential impact, and described as ‘high,’ ‘moderate,’ ‘low,’ ‘minimal’ and ‘least’ potential for impact from OA and climate change. Each category represented six separate index scores, except the middle category (low), which only included four scores. Categories were not interpreted as absolute descriptors of the risk posed to the provinces, rather they describe relative levels of potential for impact within the Atlantic Canadian region.

$$P_{P}=\frac{\left(I_{P}+Vuln_{P}\right)}{2}$$ Eq 8

Where *P* is the potential impact index score, *I* is the normalised exposure score (Eq 3) and *Vuln* is the vulnerability score (Eq 7) for province *P*.

## Results

### Species

**Fig 5:**
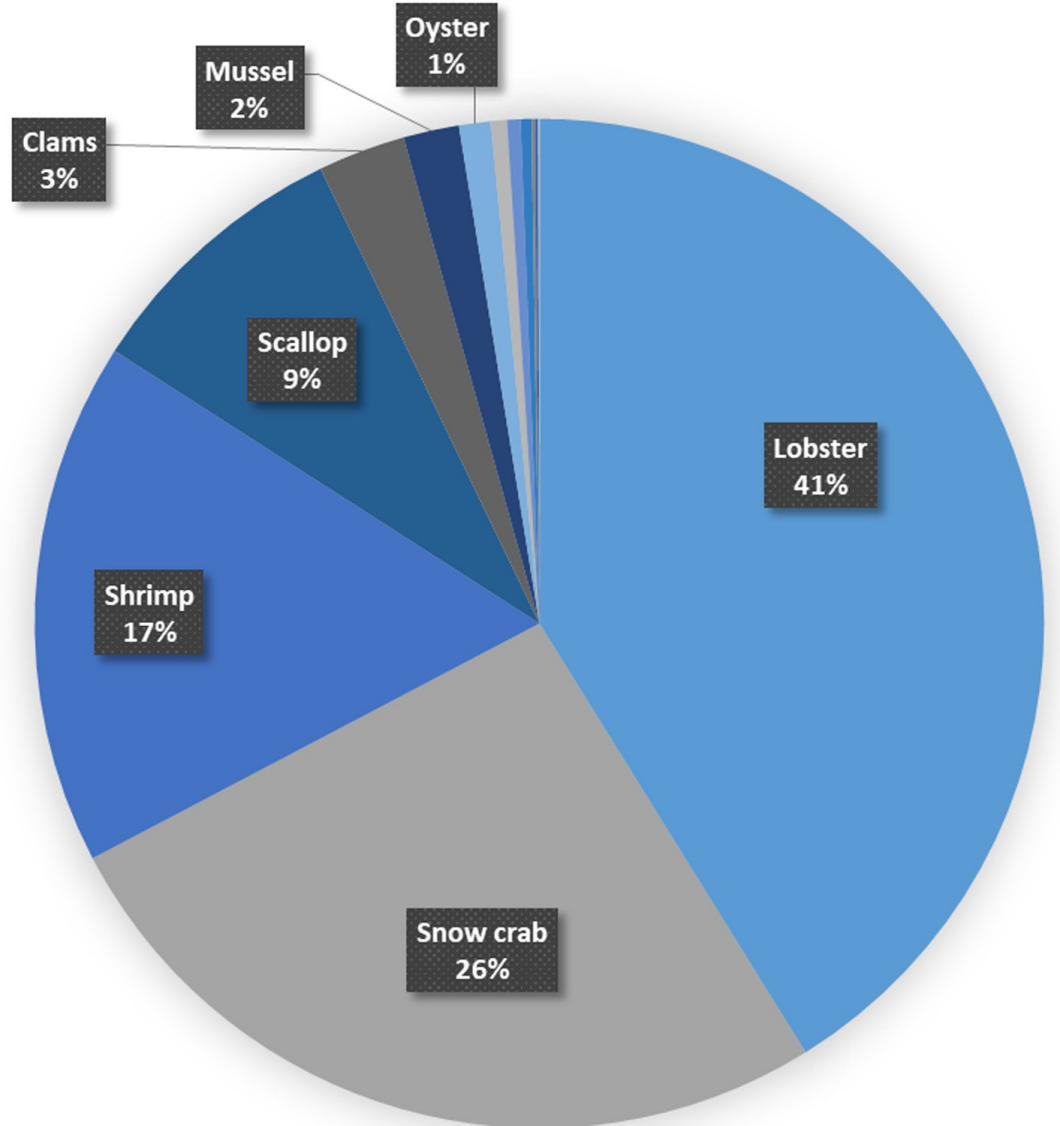
Breakdown of total shellfish average annual landings value. Data represent the average annual value from 1991–2010. Top seven most valuable species are indicated by data labels. The remaining shellfish species include other crab species (i.e., not snow crab), sea urchin, whelks and sea cucumber as well as ‘other shellfish,’ which together amount to 1.5% of the total value. Note that ‘clams’ refers to several species, however Stimpsons’ Surf Clam is by far the largest fraction of this group.

Shellfish make up nearly half of the total annual landing weight in Atlantic Canada. Furthermore, shellfish species typically receive a higher value per tonne than finfish, such that shellfish landings make up nearly three quarters of the total annual fisheries value (Fig 1 and S3 Table). Within shellfish landings, crustacean species are dominant, with northern shrimp (*Pandalus borealis)*, snow crab (*Chionoecetes opilio)* and American lobster (*Homarus americanus)* accounting for over 60% of the total shellfish landed weight (30%, 21%, and 12% by weight respectively; and 17%, 26% and 41% by landings value, Fig 5). Nonetheless, some mollusc species also contribute substantially to landings, with sea scallop *(Placopecten magellanicus)*, Stimpson’s surf clam (*Mactromeris polynyma)*, eastern blue mussel (*Mytilus edulis)* and American oyster (*Crassostrea virginica*) representing 19%, 7%, 5%, and 3% of all shellfish landings by tonnage, respectively (9%, 3%, 2%, and 1% of total shellfish value, Fig 5). Remaining shellfish species that are harvested commercially combine to make up about 5% of the total shellfish landed weight (and less than 2% by value). Due to their significant contribution to the total commercial fisheries production in Atlantic Canada, changes in landings for the above-identified species would represent a significant change for the value of the whole region’s fisheries.

### Biophysical results

#### Modelled ocean acidification impacts

**Fig 6:**
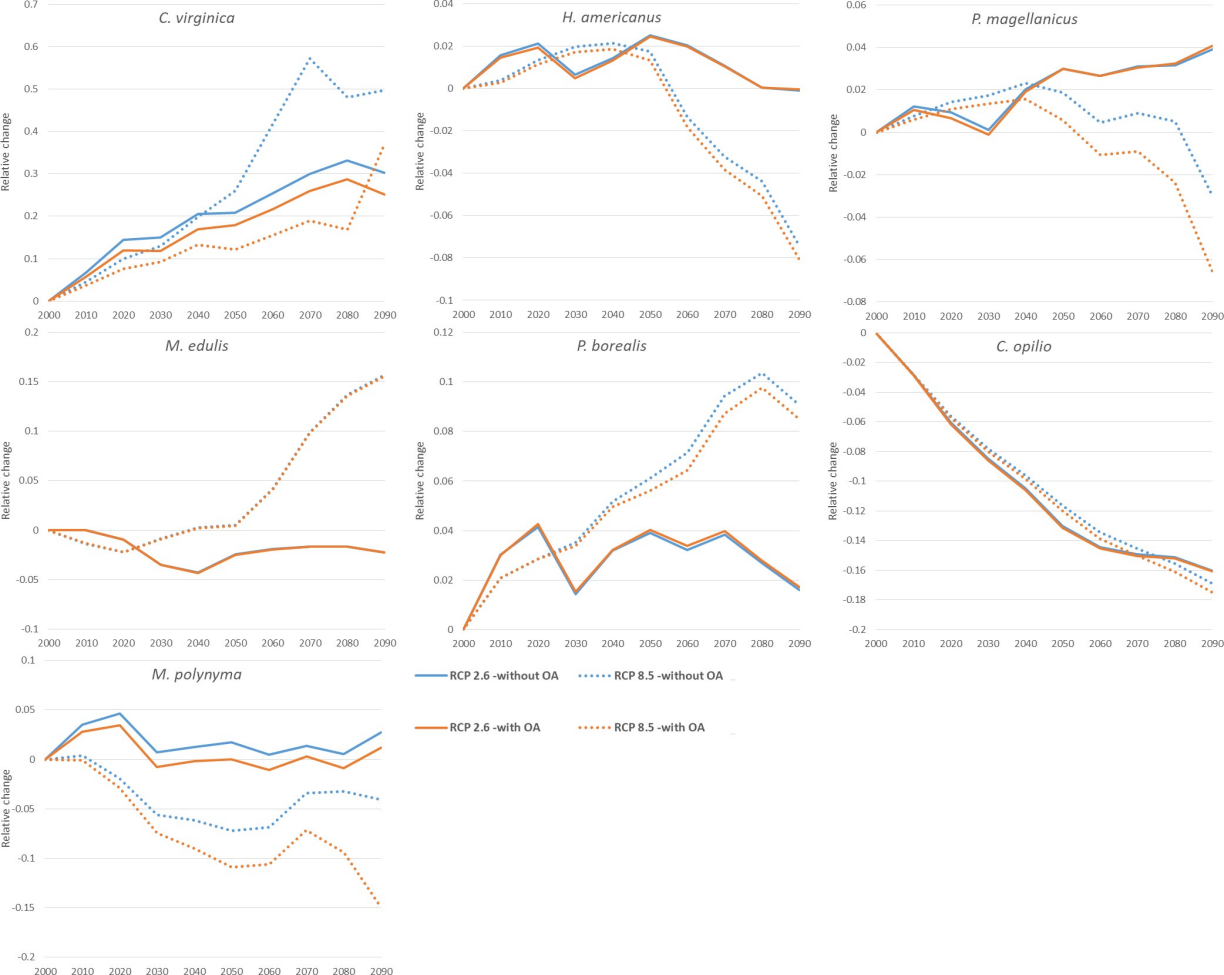
Comparison of model outputs with OA (red) and without OA (blue) effects through the century, under both climate scenarios (RCP 2.6 as solid lines, RCP 8.5 as dotted lines) for whole Atlantic Canadian EEZ. Values are relative changes in catch potential. Note different axes between sub-plots. (S1 Fig. contains relative changes in distribution for both ‘with OA’ and ‘without OA’ treatments). Lines represent multi-model medians from outputs of three earth system models (GFDL, IPSL, MPI).

When the model outputs ‘with OA’ were compared against the outputs ‘without OA’ the overall trends in landing changes were similar. However, the OA effects in all scenarios for all species moderated increases and exacerbated declines (Fig 6). The implementation of OA in the model lead to mollusc species being more strongly influenced (eastern blue mussel were an exception where the treatments were nearly indistinguishable, possibly due to low overall abundance or the eastern blue mussel physiology in the model being more affected by other environmental factors). For crustacean species the ‘with OA’ treatment tracked the ‘without OA’ treatment more closely, but were nonetheless seen to be somewhat lower throughout the simulations. Under the RCP 8.5 scenario the OA impacts were more pronounced than under the RCP 2.6 in all species (except eastern blue mussel) (Fig 6).

#### Atlantic Canadian regional changes

**Fig 7:**
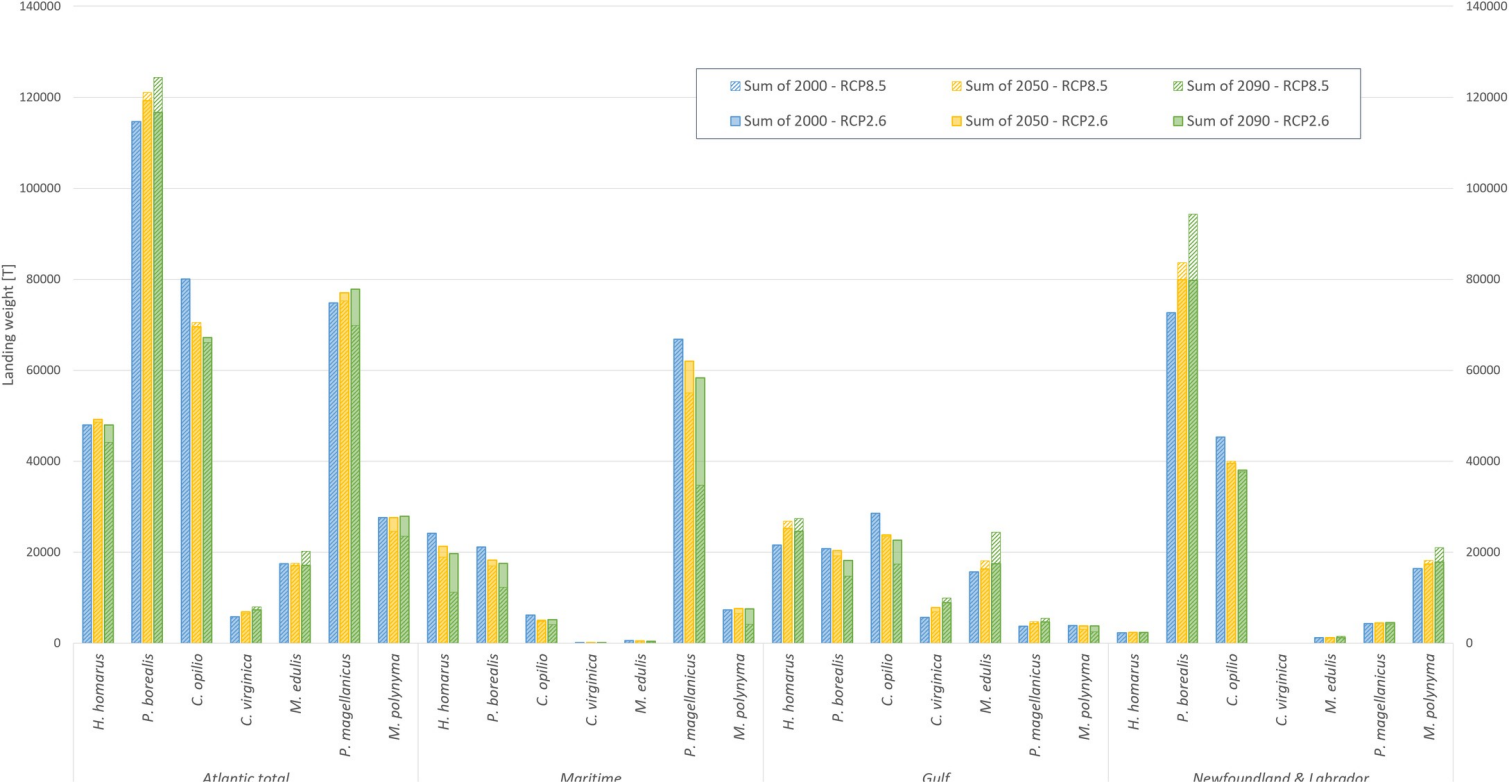
Changes in landing weight for projection scenarios for each species in the three DFO management areas and across the total Atlantic region. Each set of bars from left to right presents the year 2000 catch weight (pale blue), 2050 projected catch (yellow), and 2090 projected catch (green). Sections of each bar with diagonal hashes denote the RCP 8.5 climate scenario projections; the solid (semi-transparent) sections indicate the RCP 2.6 projections. The projected changes for the total regional landings are based on the relative change calculated across the whole study region and therefore do not present the sum of the projected landings from the individual management areas. DBEM relative change projections as percent change for each species in each time-step/climate scenario are available in S1 Table.

Across the whole Atlantic Canadian region, change in the cumulative net landings in 2090 were anticipated to be relatively neutral across the seven assessed species, regardless of the climate scenario (Fig 7). The largest relative changes were predicted for species with the lowest total landing weights in 2000: American oyster, eastern blue mussel and Stimpson’s surf clam all have changes exceeding 15% (positive for oyster and mussel, negative for clams) under RCP 8.5, but all have region-wide annual landings under 30,000 tonnes (Fig 7). Thus, relatively large percent changes resulted in comparatively small absolute changes in landings for these species. In terms of absolute change at the regional scale, northern shrimp were projected to experience the largest absolute increases in production by the end of the century (+2,000T to +10,000T for RCP 2.6 and RCP 8.5, respectively, out to 2090). However, since northern shrimp represented the highest landing weight species in 2000, the relative gains are modest (2 and 8% for RCP 2.6 and RCP 8.5, respectively). In contrast to both of these scenarios, snow crab had both high relative change and high absolute landings, with a predicted decline of 16–17% (RCP 2.6 and RCP 8.5, respectively) by 2090, on top of the second highest year 2000 landed weight (~80 000 T) (Fig 7).

Results of the DBEM under the RCP 8.5 emissions scenario tend to show more extreme changes in potential future landings, whether positive or negative, than the predictions under the RCP 2.6 scenario (Fig 7). Similarly, by the end of the century (2090) changes were generally expected to be larger than the middle century (2050) (Fig 7). There were some instances where these trends were reversed (e.g., sea scallop across the entire region—(Fig 7)); however, this generally only occurred when projected impacts were very small. Relative changes that are very small, especially when the actual landing weights are also low, are best interpreted as no change or insignificant change. Small relative changes predicted by the model may be the result of interannual variability in the underlying climate models. Nevertheless, for some of the highly landed species (e.g., northern shrimp), even a small percent change implies a sizable change in tonnes landed.

When all seven species are considered in combination across the entire region out to 2090, total landings are projected to decline, if very slightly. Under the RCP 2.6 scenario a total loss of 6,400 tonnes is predicted relative to year 2000 landings (representing a 1.8% reduction in tonnage), and for the RCP 8.5 scenario the total projected loss nearly doubles to 12,200 tonnes relative to year 2000 landings (representing a 3.3% reduction in total landings) (Fig 7). In both climate scenarios this was mainly driven by the declines in snow crab, with the RCP 8.5 scenario results compounded by more substantial losses in sea scallop and Stimpson’s surf clam (Fig 7).

**Fig 8:**
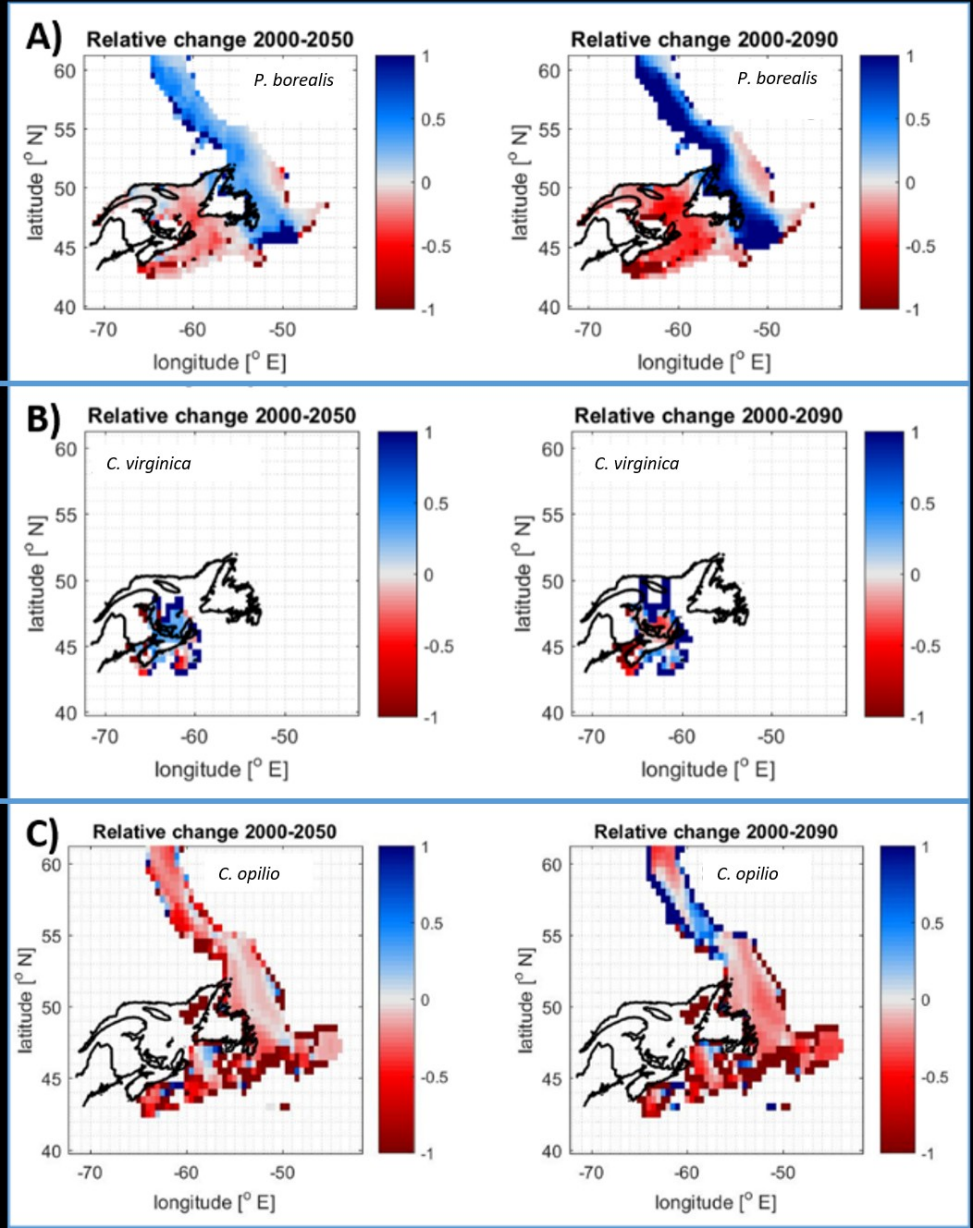
Relative changes in distribution for selected commercially targeted shellfish species. Northern shrimp (a -top), American oyster (b—middle) and snow crab (c—bottom), for 2050 (left) and 2090 (right) under RCP 8.5, highlighting south to north trend of changing species distributions. Colour scales indicate relative change predicted by DBEM for each half-degree latitude by half-degree longitude cell and do not indicate absolute values. Darkest shades (in both directions) correspond to cells with low absolute values, where small absolute changes result in large relative changes (e.g., a shift of 1T to 2T results in a 100% increase, while a change of 10T to 15T only leads to a 50% increase). Changes that exceeded +100% were set to 100% to maintain coherent colour scales. See S1 Fig for baseline DBEM distributions and figures for other species and climate treatments.

While the cumulative changes across the region were minimal, at the sub-regional scale different patterns emerged. Latitudinal gradients appeared to be the main driver of species distribution with an overall northward trend apparent for most species (Fig 8; see also S1 Fig). Overall, the Maritime management area is expected to see losses for most species (except Stimpsons’ surf clam, which experienced minimal change in all scenarios). While in the Gulf and Newfoundland and Labrador management areas a mix of changes is anticipated, with overall increases slightly outweighing declines (Fig 7).

#### Maritime management area

The three species in the Maritime management area with greatest landing weights in 2000 (American lobster, northern shrimp, and sea scallop) were all projected to decline under the quartet of exposure projections. Declines were more extreme in 2090 and under the RCP 8.5 climate scenario (Fig 7). American lobster is projected to experience the most significant loss in landings (54%). Slightly lower levels of decline were projected for sea scallop (48%) and northern shrimp (42%). Importantly, the projected declines for these commercially important species under the RCP 8.5 scenario are roughly double (or more than) the declines projected under the RCP 2.6 scenario (lobster 29%, scallop 13% and northern shrimp 17% in 2090, relative to 2000) (Fig 7).

The remaining species contributed relatively little to the Maritime management area landings and presented variable changes in their respective landings. Under the RCP 2.6 scenario snow crab had the most extreme relative decline (21%) but the loss was somewhat smaller under RCP 8.5 (16%). Even so, this species contributed a comparatively small portion of the total landings to the Maritime management area in 2000 (Fig 7). Eastern blue mussel landings also declined under all treatments, but this species was nearly non-existent in the baseline data. The two species (American oyster and Stimpsons’ surf clam) that had potential gains in landings also represent comparatively low total landings for the region. American oyster landings in the area are negligible with only ~140 tonnes of production in 2000, thus the ~10% increase predicted under RCP 8.5 for 2090 amounts to an approximate gain of 15 tonnes (Fig 7). Stimpsons’ surf clam landings change negligibly under all treatments.

#### Gulf management area

Care should be taken when interpreting model projections for the Gulf management area, as its semi-enclosed nature with substantial freshwater input from the Saint Lawrence River means it is highly influenced by processes that are not well constrained by the global climate models which inform the DBEM. Given the exploratory nature of this assessment as a starting point for anticipating future fisheries impacts in Atlantic Canada, the DBEM outputs were treated as a first approximation of potential future scenarios.

American lobster, eastern blue mussel, American oyster, and sea scallop were all projected to increase production moderately in the Gulf management area for 2090 and under the RCP 8.5 scenario (Fig 7). Eastern blue mussel had the largest relative change, increasing 68% under RCP 8.5 by 2090 (12% under RCP 2.6) (Fig 7). Notably, the Gulf management area encompasses nearly all of the current mussel and oyster production in Atlantic Canada (mainly in the form of aquaculture). Therefore, changes in production in this management area drives changes for the whole Atlantic Canadian region for these species.

Snow crab landings in the Gulf management area appeared to decline to zero very rapidly from nearly 30,000 tonnes in 2000 (representing ~35% of total snow crab landings in all of Atlantic Canada). This occurred because in two of the three climate models (GFDL and IPSL) and hence the median dataset–the DBEM-modelled distribution in the Gulf in 2000 was minimal (S1U–S1X Fig), and amounted to less than 1% of the total predicted landings for the whole of the Atlantic Canadian region. When the modelled projections of this small initial distribution fell to zero, it resulted in relative change of -100%. The outputs based on the third climate model (MPI) predicted higher initial abundances and therefore less extreme declines of snow crab in the area (reductions of 20% and 39%, for RCP 2.6 and RCP 8.5, respectively). Therefore, a mean of the three climate model impacts was adopted for the analysis of this species. Under this approach snow crab is projected to decline in the Gulf management area by 17–18% in 2050 (RCP 2.6 and RCP 8.5, respectively) and 21–39% by 2090 (RCP 2.6 and RCP 8.5, respectively) from the 30,000 tonnes landed in 2000 (Fig 7).

#### Newfoundland and Labrador management area

The Newfoundland and Labrador management area not only encompasses the largest area of marine waters (**Fig 1**) but also has some of the highest landings, by weight, with over 50% of the total Atlantic Canadian catch for northern shrimp, snow crab and Stimpson’s surf clam in 2000 (Fig 7). The patterns for northern shrimp and Stimpson’s surf clams were similar for both scenarios and time-steps, albeit at different scales, as the shrimp landings were more than four times that of the clam. For both species under the RCP 2.6 scenario there are modest projected increases in landings by 2050 (10% and 6% for shrimp and clams, respectively). However, under this scenario projected production to the end of the century remains relatively unchanged, with shrimp staying at 10% above 2000 levels, and clams increasing slightly to 9% over 2000 landings. In contrast, under the RCP 8.5 scenario both species continue to increase in production through to 2090 (Fig 7).

As observed in the other management areas, snow crab is also projected to decline in the Newfoundland and Labrador management area. The losses are similar between the RCP climate scenarios, while the landings for 2090 declined slightly from the 2050 values, from 13–12% in 2050 (RCP 2.6 and RCP 8.5, respectively) to 16% in 2090 (for both climate scenarios).

The area is expected to see minor changes for lobster, scallop and mussel landings under the quartet of projections, with mussel production presenting an exception under RCP 8.5 at the end of the century with an expected increase of 21% by 2090 under RCP 8.5. Nevertheless, all three species represent minor fractions of the region’s landings and have much higher production values in the other two management areas.

### Impact assessment results

#### Exposure

Scaling the DBEM-modelled changes in landings by species value in 2000 resulted in an array of indicator scores representing the changes in the seven assessed species (Table 2). In most quartets of exposure scores, the 2090 time-step had higher exposure than the 2050 time-step—NL was an exception to this trend under the RCP 8.5 scenario (Table 2). The RCP 8.5 climate scenario drove increased landings for some fisheries, thus the scores corresponding to this climate scenario were higher for both the Gulf and the Newfoundland and Labrador management areas. Conversely, they were lower in the Maritime management area (and for the region as a whole) (Table 2).

The Maritime management area was projected to experience losses across its quartet of exposures with the 2090 time-step and RCP 8.5 climate scenario resulting in losses of nearly 50% of the value-scaled landings (Table 2). The scores for this management area were relevant to the NB-mar and NS-mar provincial sub-sections. Conversely, the Gulf area scored slightly positive in all scenarios, however 2090 for both climate scenarios had slightly reduced scores compared to 2050 (Table 2). The Gulf management area scores were applied to PEI and Que as well as the Gulf sub-sections of NB and NS. Notably, the Newfoundland and Labrador management area scored (slightly) negative under the RCP 2.6 scenario for both time-steps, but had positive scores under the RCP 8.5 scenario, largely due to the stronger gains in shrimp for the area under the latter treatment (Table 2). The Newfoundland and Labrador management area was only applied to the province of NL. The score as calculated for the whole Atlantic region was slightly negative under the quartet of treatments, although the region as a whole was not included in the final index calculation (Table 2).

#### Social vulnerability

The scores for each of the indicators that contributed to vulnerability are presented in Tables 3 and 4. The scores are presented as both the raw scores before normalisation of the data, as well as the normalised indicator scores which were used to calculate the final impact index scores. The cumulative vulnerability scores (i.e., combinations of sensitivity and adaptive capacity) are presented in the final risk index in Table 5.

**Table 3:** Indicators scores for sensitivity. Columns 2–3 present absolute scores for each province while columns 4–5 present the corresponding normalised scores as used to calculate provincial sensitivity (column 6). Higher sensitivity scores contribute to higher potential impact. The sensitivity scores were combined with the adaptive capacity scores (as indicated in Fig 4) in Table 4 to define vulnerability for each province (Table 5).

Province	Shellfish harvesting employment / capita^(a)^	Shellfish value / GDP^(b)^	Normalised shellfish employment score	Normalised shellfish value score	Sensitivity
**NB**	0.013	0.008	0.21	0.18	0.19
**NL**	0.044	0.019	0.76	0.48	0.62
**NS**	0.021	0.020	0.36	0.50	0.43
**PEI**	0.058	0.040	1.00	1.00	1.00
**Que**	0.001	0.001	0.00	0.00	0.00

Data references:

(a)**[45–50,54,55];**

(b)**[20,44]**

**Table 4:** Indicators scores for adaptive capacity. Columns 2–4 present absolute scores for each indicator. Normalised indicators were reversed (i.e., subtracted from 1.0) (columns 5–7), so that low indicator scores contributed to higher potential. The combined adaptive capacity score (last column) is the average of the reversed, normalised indicators. Adaptive capacity scores were combined with the sensitivity scores (as indicated in Fig 4) in Table 3 to define vulnerability for each province (Table 5).

Province	[*Reciprocal*] Un-employment / national un-employment^(a)^	Post-secondary education / national post-secondary education^(b)^	Aquaculture development / mollusc production^(c)^	(Reversed) Normalised un-employment score	(Reversed) Normalised education score	(Reversed) Normalised aquaculture score	(Reversed) Adaptive capacity
NB	0.092	0.46	0.51	0.25	1.00	1.00	0.75
NL	0.059	0.47	0.68	1.00	0.88	0.74	0.87
NS	0.098	0.53	1.14	0.11	0.00	0.00	0.04
PEI	0.077	0.49	1.05	0.60	0.62	0.14	0.45
Que	0.103	0.52	0.80	0.00	0.18	0.54	0.24

Data reference:

(a)**[51]**;

(b)**[52]**;

(c)**[20,56]**

**Table 5:** Impact index components and scores. Components are presented as final normalised scores. The exposure scores are presented in columns 3–4 as quartets of climate scenario and future time-step. The vulnerability scores (column 5) are the average (i.e., equally weighted combination) of sensitivity (Table 3) and adaptive capacity (Table 4) as indicated by Fig 4. The final index score quartets (column 6–7) are the average (i.e., equally weighted combination) of the relevant exposures and vulnerabilities. The final two columns indicate rank for each unique potential impact score: 1-high, 2-moderate, 3-low, 4-minimal and 5-least potential for impact (all categories except 3-low, represent 6 unique scores– 3-low only represents 4 unique scores).

Province	RCP	Exposure 2050	Exposure 2090	Vulnerability	Impact 2050	Impact 2090	Impact Ranking	
NB-gulf	2.6	0.05	0.09	0.47	0.26	0.28	4	3
	8.5	0.00	0.06		0.24	0.27	4	4
NB-mar	2.6	0.32	0.42		0.40	0.44	2	1
	8.5	0.48	1.00		0.48	0.74	1	1
NL	2.6	0.15	0.17	0.75	0.45	0.46	1	1
	8.5	0.10	0.02		0.42	0.38	2	2
NS-gulf	2.6	0.05	0.09	0.23	0.14	0.16	5	4
	8.5	0.00	0.06		0.12	0.15	5	4
NS-mar	2.6	0.32	0.42		0.28	0.33	4	3
	8.5	0.48	1.00		0.36	0.62	3	1
PEI	2.6	0.05	0.09	0.73	0.39	0.41	2	2
	8.5	0.00	0.06		0.36	0.39	3	2
Que	2.6	0.05	0.09	0.12	0.08	0.11	5	5
	8.5	0.00	0.06		0.06	0.09	5	5

Quebec scored very low in nearly all of the indicators contributing to vulnerability (Tables 3 and 4). The aquaculture indicator was the only exception due to the relatively small shellfish aquaculture sector in the province (Table 4). The low scores are likely a result of the social structure of Que being unique among the assessed provinces in many respects. Industry in the province is less dependent on natural resources and its population is an order of magnitude larger than any other province in the assessment–the population of Que is more than three times larger than the four other provinces combined. Conversely, Que has the smallest fisheries sector of the five provinces, as such the industry has a very low relevance to the economy and population at large.

Nova Scotia had the second lowest cumulative vulnerability score (Table 5), largely driven by strong adaptive capacity indicators (especially aquaculture and education) (Table 4). Despite having the lowest education and aquaculture indicator scores, and a consequently low adaptive capacity, NB ranked third in vulnerability due to a relatively low reliance on shellfish, and hence a low sensitivity score (Tables 3 and 4).

PEI and NL were the most socially vulnerable provinces (Table 5). PEI’s vulnerability derived from having the highest sensitivity due to a comparatively small population with a relatively high dependence on shellfish fisheries (Table 3). Additionally, PEI had moderate scores for two of the three adaptive capacity indicators, with relatively low education and high provincial unemployment (Table 4). The strong aquaculture sector in PEI was not sufficient to offset the other indicators. NL had the lowest overall vulnerability score (Table 5). However, provincial unemployment was the only indicator where it earned the lowest provincial score (Table 4). Rather, the low overall vulnerability score was driven by generally weak scores across all the indicators, with an absence of any strong adaptive capacity or low sensitivity indicators to counterbalance the low scores.

#### Impact index scores

Potential impacts from OA and climate change are anticipated to be highest where high exposure and high vulnerability coincide [17,18,57] (Table 5). Since there was only a single vulnerability score for each province, while the exposure was represented by a quartet of scores, the patterns within a given province’s quartet of index scores mirrored the patterns in the exposure: 2090 generally had higher potential impacts than 2050. Under the RCP 2.6 scenario the index scores tended to be higher due to more exaggerated gains in landings (and hence lower potential for impact) projected under RCP 8.5. The index scores coinciding with exposure to the Maritime management area (NB-mar and NS-mar) were the exceptions to this and had much higher potential impacts under RCP 8.5 (Table 5).

Relatively high exposure scores were only achieved for the two provinces that have fisheries in the Maritime management area (i.e., NS and NB). Within these, NS had fairly low vulnerability scores, while NB scored mid-range in vulnerability (Table 5). The highest single exposure score was anticipated for 2090 under the RCP 8.5 climate scenario (Tables 2 and 5). The effect of this exposure treatment was strong enough that NB-mar and NS-mar scored the two highest potential impact scores. However, the low vulnerability in NS offset the exposure for the remaining Maritime management area quartet, and the remainder of the NS-mar impact scores were ranked low to minimal potential impact (Table 5). The higher vulnerability in NB meant the province was more directly influenced by the exposure indicator and consequently ranked high for potential impact in three of the four of exposure scenarios (Table 5).

Due to the occurrence of high vulnerability and moderate exposure, the NL index quartet ranked high to moderate, with the RCP 2.6 climate scenario exposures scoring higher than the unmitigated scenario (RCP 8.5) (Table 5).

The remaining provincial units (NB-gulf, NS-gulf, PEI, and Que) were all adjacent to the Gulf management area and therefore had very low normalised exposure scores (Table 5). PEI mainly ranked in the moderate potential impact category due to high vulnerability, with only the 2050 –RCP 8.5 exposure ranking as low potential for impact (Table 5). The gulf sub-divisions of NB and NS (NB-gulf and NS-gulf) ranked from low to least potential for impact. Out of the two, NB-gulf had slightly higher overall risk due to higher social vulnerability. Quebec, which had the lowest vulnerability along with the low exposures associated with the Gulf management area, predictably earned the lowest index scores (Table 5).

#### Sensitivity testing

In order to test the robustness of the framework construction (i.e., Fig 4), alternate indicator weighting and indicator aggregating steps were considered. Most of the alternate orientations mainly re-arranged the mid-level index values (i.e., the moderate to minimal impact categories). Although there were some notable adjustments in the higher potential impact categories. In all the tested weighting orientations Que maintained the lowest scores.

Weighting each individual indicator equally (i.e., setting each indicator to contribute equally to total index scores) rather than equal within each branch in the framework, caused the effect of the exposure indicator to be diluted so that the total scores were more similar to the provincial vulnerability scores. In this weighting system NL and PEI scored the highest index scores, followed by NB (S2 Fig and S4 Table). NS-mar and NS-gulf filled out the minimal and least potential impact categories.

When exposure, sensitivity and adaptive capacity were weighted equally (i.e., sensitivity and adaptive capacity were kept separate, rather than combined to form vulnerability), in a framework configuration which more closely resembled the orientations used in vulnerability assessments such as [11,14], NL and PEI again ranked higher due to their weak scores in the sensitivity and adaptive capacity (S2 Fig and S4 Table). In contrast, other risk assessment frameworks have used hazard, exposure and vulnerability as three equally weighted components of risk [15,17]. However, in this assessment, the hazard was effectively incorporated into the exposure term. To test the effect of including an equally weighted hazard component, the exposure scores were weighted twice as heavily as vulnerability. Under this weighting system NS-mar and NB-mar scored higher with some of their scores moving up a rank. Conversely, NL along with the NS-gulf and NB-gulf scored relatively lower (S2 Fig and S4 Table). Since the indices resulting from the alternate orientations emphasised either the vulnerability or the exposure indicators, the equal weighting system used throughout the analysis presented the most neutral approach.

## Discussion

### Fisheries changes in Atlantic Canada

Temperature-driven changes are expected to shift marine species’ ranges poleward [e.g., 32,40,58]. Six of the seven species considered in this study are currently near the middle or even at the southern extent of their historic natural range in Atlantic Canada (i.e., American Lobster, northern shrimp, snow crab, sea scallop, eastern blue oyster and Stimpson’s surf calm [59]). It was, therefore, unsurprising that the DBEM predicted changes in species distributions in the study region resulted in the southernmost management area (i.e., the Maritime management area) experiencing declines for most species under the quartet of exposure scenarios (Fig 7). Meanwhile, the Gulf along with the Newfoundland and Labrador management areas were predicted to see modest increases in landings for most species under most of the treatments (Fig 7).

Snow crab, which requires very cold bottom waters [60], was predicted to see substantial declines in all scenarios and management areas (Fig 8C). This was consistent with other recent assessments of marine species distributions in Atlantic Canadian waters under warming ocean temperatures [61]. Due to its significant contribution (both in value and tonnage) to overall fisheries landings in Atlantic Canada, the region-wide anticipated decline in future snow crab landings impacted the cumulative fisheries changes across the management areas (Fig 7).

American oyster was the only assessed species that is currently near the northern limits of its distribution in Atlantic Canadian waters. At present, landings for the fishery are relatively small (Figs 5 and 7), but overall, the region is expected to become better suited for the species. With most of its current production deriving from aquaculture, the industry could be in a position to take advantage of the improving habitat suitability in the region. Enhanced aquaculture production of oyster could help to partially compensate for losses anticipated of other species, especially in the southern reaches of the region. However, current American oyster production rates are orders of magnitude lower than most of the other assessed species, so any mitigation potential for losses are likely to be highly localised.

### Potential impacts and social vulnerability

The communities in NB that rely on fisheries in the Maritime management area were found to be most at risk to OA and climate change. Though, the bulk of NB shellfish production occurs in the Gulf management area and is therefore not exposed to the changes in the Maritime management area. Nonetheless, the sub-section of the province reliant on the Maritime management area should be considered as an area for pro-active responses to potential changes in fisheries driven by OA and climate change. Conversely, the NB-gulf sub-section did not appear to be a high risk area, and scored minimal potential for impact in all of four of the exposure scenarios. The contrasting potential impact levels within the province may present an opportunity to locally mitigate impacts in the Maritime management area dependent communities, as production and harvesting activity could be shifted to the potentially less exposed management area. Such shifts would certainly have to take the potential consequences of increasing fishing capacity in the Gulf management area into account. The within province gradient in future resource accessibility may present a microcosm of the Atlantic Region as a whole, where declines in one location are, to an extent, counterbalanced by gains in another.

The provinces of PEI and NL were also at generally higher risk from OA and climate change, mostly ranking in the high to moderate potential impact categories. The potential impacts in these provinces were predominantly driven by social vulnerability rather than changes in access to the fisheries themselves. Many of the vulnerability factors (such as education and unemployment) occur at local scales and are therefore more immediately actionable by provincial decision-makers. As such, these provinces may be able to more directly pursue social and economic shifts to reduce risk from OA and climate change.

Overall, NS scored a single high potential impact score (in 2090 under RCP 8.5) for the Maritime management area; otherwise the province ranked low to least potential for impact. In spite of being highly exposed in communities which depend on the Maritime management area, Nova Scotia’s relatively low vulnerability suggests that the province will generally be more capable of responding to the changes in shellfish production driven by OA and climate change than other provinces in the region. However, it is possible that communities within the province have much less capacity to adapt than the provincial statistics imply. As with most of the other provinces in the region, NS has a relatively high rural population and many small coastal communities with a high dependence on fisheries [23,24]. Given the relatively strong declines anticipated for the Maritime management area, a more localized investigation may be warranted for this province.

Lastly, Que appears to be largely unthreatened by OA and climate change impacts on fisheries. The province as a whole is not vulnerable to changes in shellfish production, and under the DBEM projections the comparatively small shellfish harvest was not highly exposed to change.

### Responding to risk

To reduce exposure to OA and climate change a global scale response is required. That said, some localised factors that amplify OA (e.g., eutrophication) can be acted upon at more confined scales [62]. In order to reduce impacts from OA and climate change in Atlantic Canada (and the rest of the planet), global efforts need to be made to reduce carbon emissions [e.g., 1,39,63]. Local policies can, and should, be enacted to reduce emissions and to contribute to reductions in atmospheric CO_2_ concentrations. However, action directly responding to anticipated changes in resource availability will also be important for mitigate the impacts on coastal communities and economies.

Factors affecting social vulnerability can be acted upon locally by regional managers and decision-makers to reduce risk. Although this will not reduce the ecological impacts caused by OA and climate change, it can help to reduce the impacts felt by human communities. In the case of vulnerability related to Atlantic Canadian shellfish fisheries, there are multiple opportunities for mitigating actions. While education levels were near the national rate for most of the provinces (Table 4), efforts to improve education rates could alleviate some of the social vulnerability in the region by opening more employment opportunities to the populations. The education statistic considered here was a broad indicator of overall education in the provinces, however, more targeted education programs regarding the future of the affected fisheries (for both increasing and decreasing abundances) could greatly improve the adaptability of the harvesters and communities which rely on them and help to promote sustainable long-term harvests [64]. Similarly, addressing provincial unemployment rates, which were above the national levels in all of the provinces, could greatly reduce vulnerability to potential lost employment in declining fisheries. Improvements in either (or both) of these indicators should also improve provincial adaptive capacity regarding a range of potential climate change driven impacts.

The third adaptive capacity indicator, aquaculture, is more case specific but also presents a direct way to respond to OA impacts on shellfish production in the region. Current aquaculture production of shellfish in Atlantic Canada is mainly dependent on wild populations for recruitment and hatchery production in the region is limited [27]. However, investment in hatchery infrastructure could greatly improve the region’s ability to maintain production of mollusc species in the face of falling pH or irregular natural recruitment. Adaptation of hatchery procedures is already being used to mitigate low pH events on the Pacific Coast of Canada and the United States; the expertise developed there could be leveraged to support Atlantic production [8,53]. It is worth noting that while improving shellfish aquaculture may help to maintain production levels, it is not a direct substitute for loss of livelihood from decreased capture fisheries. Fish harvesters may be unlikely to actively pursue a transition to aquaculture production. Furthermore, in terms of actual employment aquaculture is a more efficient method of production, by tonnage of product, and typically requires fewer people to produce equivalent harvests. Nonetheless, as a factor to reduce overall impacts from changes in fisheries, aquaculture (with hatcheries) is an opportunity worth further consideration in the region.

The indicators related to sensitivity are somewhat more difficult to address because they are more directly tied to the fishing industry and are ingrained in the social and cultural identity of the region. Furthermore, DFO acknowledges that overcapacity already exists in many Atlantic Canadian fisheries [23]. Incrementally reducing overall fishing effort could help to limit sensitivity to changes in the fisheries and support overall conservation targets [23,65]. To be a viable option, this would first require that alternative employment opportunities existed. Diversifying harvests is another mechanism that can be enacted to dampen losses in any one fishery [23]. Diversity of mollusc species harvested has previously been used as a component of adaptive capacity in their assessment of vulnerability to OA in the United States [14]. However, many of the commercial harvesters in Atlantic Canada already target multiple species [23]. While this may ultimately indicate that the provinces are less sensitive to changes than this assessment implies, it also means that this aspect will be difficult to improve upon as a method to reduce vulnerability for the region. Developing other industries and factors supporting provincial economies could reduce the relative importance of fisheries in the region and thereby reduce sensitivity related to changing fisheries.

A finer scale assessment is also potentially highly relevant for addressing potential impacts in Atlantic Canada. Much of the region is made up of small communities with much more localised economic and social factors than is represented by the provincial scale statistics [23,24]. In some counties, employment in shellfish fisheries is much higher than the provincial rates imply–especially when additional steps of the supply chain are included. For example, lobster fishing area (LFA) 34 is by far the most productive area for American lobster in the Maritime management area [66]. Following an approximation that counties rely most heavily on adjacent management areas, this implies that counties such as Digby, Yarmouth and Shelburne could be much more sensitive to changes in that fishery than other NS counties. Moreover, LFA 34 corresponds to the area with some of the most substantial DBEM projected losses for lobster landings (S1E–S1H Fig). Future assessments at a finer social scale might be necessary to identify these potentially more at-risk communities. Following the conclusions of this study, NB and NS would be strong candidates for future fine-scale assessment, as these provinces were most exposed to changes in fisheries, thus highly vulnerable communities in these provinces could be among the most at risk in the region. Relatedly, future assessments could include more detailed economic models to better account for how markets may respond changes in the industry.

More broadly, future research into socioeconomic implications of OA and climate change will benefit substantially from better understanding species and ecosystem responses to OA and other climate change stressors, especially including how responses to these stressors will interact. Conclusions could also be improved with more detailed biophysical models. To this end improving the resolution and incorporating finer scale ocean currents and processes into models, such as the DBEM used here, would allow for more nuanced estimates of social impacts and more specific predictions of where and how fisheries will potentially be most affected. Consideration of additional changes to ecosystem functions, such as increased rates of harmful algal blooms, will also benefit future estimates of potential impacts on fisheries.

Additionally, the OA impact level in the iteration of the DBEM used in this research was based on the mean effect from two meta-analyses [3,4]. The OA driven losses could be much more severe than anticipated by the model, potentially to the point where OA might not be entirely overwhelmed by temperature driven patterns in abundance. Conversely, the OA impacts could also be much less severe and the benefits for many species in the region from thermal loading could be more pronounced. Recent efforts have explicitly tested different impact levels if OA in the DBEM [34]. Thus future socioeconomic assessments using DBEM projections may be able to examine a broader range of OA impact levels. Similarly, the OA effects in this assessment assume a linear impact from OA (i.e., as pH changes there was a linear relationship to the affected life-history traits–growth and survival). This treatment does not allow for adaptation, where species might be able to limit the effects of OA through biological mechanisms such as acclimatisation (including behavioural responses), parental effects (i.e., epigenetic effects) or evolution [e.g., 67]. However, including a simulation of adaptation in the DBEM (where the pH level is applied in relation to the previous time-step rather than the baseline level) appears to lead to negligible effects from OA in the model outputs [34].

### Conclusion

The findings of this assessment contrast with previous socioeconomic analyses of OA effects on fisheries. In previous assessments of OA impacts [11–17], projected changes in fisheries have been presented almost exclusively as declines in potential landings with subsequent impacts on societies and economies. However, the majority of these studies investigated OA impacts in isolation from other facets of climate change and did not account for potential multi-stressor impacts. This study demonstrates that accounting for additional environmental factors can allow for different, and potentially more representative, narratives to emerge. Future management decisions and mitigation plans could be better informed through analyses which account for a more complete range of future effects on resource accessibility.

Atlantic Canada is a region with an exceptionally high dependence on OA-susceptible fisheries. However, the findings presented here suggest that OA-driven declines in the region will be minor compared to temperature-driven changes in future landings (Fig 6). Nonetheless, fisheries resources in Atlantic Canada are expected to see some notable redistributions under OA and climate change over the coming century. As observed in global-scale studies this is expected to result in ‘winners and losers’ with respect to access to future fisheries [e.g., 40]. It may be tempting to view the ‘winners’ as gaining access to new production. However, it is essential to recall that perceived increases come at a cost to other regions [68]. Additionally, while OA effects may appear to be locally overwhelmed by temperature driven increases this should be considered in context and interpreted as limiting potential gains, and exacerbating declines.

Within the Atlantic Canadian region, management plans should be developed that take climate change and shifting species distributions into account. These plans should specifically address future allocation of resources when long-held access to certain high value fisheries (e.g., American lobster) might transition into new jurisdictions. Moreover, these considerations need to be extended to cooperative management of marine resources between nations. For example, net gains in Atlantic Canada may come at a cost to US states to the south.

Management plans seeking to account for changing access to resources will benefit from assessments, such as in this study, which address how and where biological changes are likely to affect human communities, and where efforts are needed to mitigate these impacts.

## Supporting information

S1 Note Additional details for DBEM. (PDF)

S2 Note Details inclusion of ‘inshore’ and ‘offshore’ fleets and special cases regarding data availability. (PDF)

S1 Table DBEM projected relative change from individual climate models for each climate scenario and both future time-steps for each species. (ODS)

S2 Table data sources for crew size estimates. (PDF)

S3 Table Annual landing value (year 2000 dollars) and weights (tonnes) for marine species in Atlantic Canada. Values for DFO fisheries species groups (finfish, groundfish and shellfish) are presented, as well as values for individual shellfish species.
(ODS)

S1 Fig DBEM projections for OA and RCP treatments. Each species is presented in four figures representing: RCP 2.6 ‘with O,’ RCP 2.6 ‘without OA,’ RCP 8.5 ‘with OA’ and RCP 8.5 ‘without OA,’ respectively. Species are presented as follows: a-d = American oyster; e-h American lobster; i-l sea scallop; m-p eastern blue mussel; q-t northern shrimp; u-x snow crab; and y-ab Stimpsons’ surf clam. Each figure includes: top left–species graphic; top right–relative change for management areas. Scale is the same for each figure; therefore, species which extend beyond 25% change in either direction go beyond the figure axes (note data separates NAFO subareas that border DFO management areas (i.e. 4R and 4Vn), line colours correspond to distribution map in bottom left). Bottom left–DBEM predicted distribution for baseline year 2000 (averaged 1991–2010). Management borders outline colour corresponds to relative change in top right panel. Bottom middle–relative change in 2050 (average 2041–2060) compared to 2000 (average 1991–2010). Bottom right–relative change for 2090 (average 2081–3000) against 2000 (average 1991–2010). Note that colour scales presented in bottom-middle and bottom-right panels are relative changes within individual data cell. An artefact of this was that cells which had zero presence in 2000 but gained presence in future time steps had an infinite increase in catch potential; therefore, all increases above 100% were presented as 100%. Relatedly, the vast majority of the cells that experience 100% increases or decreases represent small baseline inputs (as per the bottom right panel).
(ODS)

S2 Fig Alternate framework orientations for sensitivity testing. Letters correspond to sensitivity testing risk outcomes in S4 Table
(TIF)

S4 Table Risk index results for 3 alternative weighting schemes. Framework orientations schematics presented in S2 Fig.
(ODS)
