# Supplementary material for: Potential socioeconomic impacts from ocean acidification and climate change effects on Atlantic Canadian fisheries
Source: PLoS One. 2020 Jan 10;15(1):e0226544. doi: 10.1371/journal.pone.0226544 (PMC6953801; doi:10.1371/journal.pone.0226544)
Supplement: S1 Note — (PDF) [file pone.0226544.s001.pdf]

Supplementary DBEM

We provide details of the DBEM that are relevant to understand the outputs used in the subsequent ocean acidification socioeconomic analysis.

Ecophysiological models

Ocean acidification impacts were modelled with other stressors (i.e. temperature, dissolved oxygen) and uses a derived equation of the von Bertalanffy growth function (Tai, Harley, & Cheung, 2018) to determine changes in growth rate, *B*, over time, *t*:

$$\frac{dB}{dt} = HW^d - kW^b \tag{1}$$

where *H* and *k* represent the coefficients for anabolism and catabolism, respectively. The growth rate is a function of the oxygen supply (i.e. anabolism) and oxygen demand required for maintenance metabolism (i.e. catabolism). Anabolism scales with body weight (*W*) to the exponent $d < 1$. In this model, $d = 0.7$, although values typically range from 0.5 and 0.95. Sensitivity analyses showed that changes in temperature and acidity with low values of *d* (< 0.7) slightly decreases sensitivity, while larger values of *d* (>0.7) markedly increases sensitivity (Pauly & Cheung, 2017; Tai et al., 2018). The use of 0.7 is thus a conservative value as smaller values of *d* only marginally decrease sensitivity to multiple stressors and larger values of *d* only increases sensitivity. Catabolism scales linearly with (*W*), i.e. $b = 1$.

When maximum body size (W_∞) is reached and $dB/dt = 0$, solving for equation (1) results in $H = kW_\infty^{(1-d)}$. Then we can integrate equation (1) into a generalized von Bertalanffy growth function:

$$W_t = W_\infty \left[1 - e^{-K(t-t_0)}\right]^{1/(1-d)} \quad (2)$$

where *K* is the von Bertalanffy growth parameter where $K = k(1-d)$. The parameter *K* represents the rate at which maximum body size is reached.

Environmental stressors are integrated to affect metabolism using:

$$H = g[O_2]e^{-j_1/T} \quad (3)$$

and

$$k = h[H^+]e^{-j_2/T} \quad (4)$$

H and *k* coefficients as a function of the Arhennius equation, $e^{-j/T}$, where j_1 and $j_2$are equal to E_a/R where E_a (for anabolism and catabolism, respectively) and *R* are the activation energy and Boltzmann constant, respectively. Constants j_1 and $j_2$are equal to E_a/R where E_a (for anabolism and catabolism, respectively) and *R* are the activation energy and Boltzmann constant, respectively, while T is the absolute temperature (in Kelvin) (Cheung, Dunne, Sarmiento, & Pauly, 2011). Oxygen availability (i.e. dissolved oxygen concentration) and acidification (i.e. hydrogen ion concentration) affect aerobic scope changing oxygen supply *(*anabolism) and oxygen demand (catabolism), respectively. Our model assumes that multi-stressor impacts following the most viable and parsimonious current working hypothesis (oxygen- and capacity-limited thermal tolerance hypothesis), to link physiological responses to life history traits. Coefficients *g* and *h* from equations (3) and (4), respectively, were derived for each species from the average W_∞, *K*, and environmental temperature T_0 reported in the literature (Cheung et al., 2011):

$$g = \frac{W_{\infty}^{(1-d)}K}{[O_2]e^{-j_1/T}} \quad (7)$$

and

$$h = \frac{K/(1-d)}{[H^+]e^{-j_2/T}} \quad (8)$$

Changes in aerobic scope are linked to changes in the asymptotic weight (W_∞) and von Bertalanffy growth parameter K:

$$W_\infty = \left(\frac{H}{k}\right)^{1/(1-d)} \quad (9)$$

and

$$K = k(1-d) \quad (10)$$

Other parameters—e.g. asymptotic length and the length at maturity—that scale with weight can also be predicted, (Beverton & Holt, 1959).

Population dynamics models

We modelled populations using an intrinsic population growth model (Hilborn & Walters, 1992):

$$G_i = r \cdot A_i \cdot \left(1 - \frac{A_i}{KC_i}\right) \quad (12)$$

where G_i is the population growth in any given grid cell i, r is the intrinsic rate of population increase, A is the abundance, and KC is the carrying capacity. Thus, changes in abundance was modelled as:

$$\frac{dA_i}{dt} = \sum_{j=1}^{N} G_i + L_{ij} + I_{ij} \tag{13}$$

where *L* and *I* are the settled larvae and net migrated adults, respectively, from surrounding cells *j* into focal cell *i*.

Carrying capacity was modelled to change as a function of habitat suitability, *P*, with the equations (Cheung, Lam, & Pauly, 2008) :

$$KC_{t+1} = KC_t \cdot \left(\frac{P_{t+1}}{P_t}\right) \tag{14}$$

and

$$P = P(T) \cdot P(Dep) \cdot P(H) \cdot P(Ice) \tag{15}$$

where *T*, *Dep*, *H*, and *Ice* are corresponding temperature, depth, habitat type, and sea ice coverage for each cell, respectively. A major assumption of the model is that the each cell is at carrying capacity at the start of the simulation—the averaged 1951 to 1970 time period.

References for S1 Note:

Beverton, R. J. H., & Holt, S. J. (1959). A review of the life span and mortality rate of fish in nature and the relation to growth and other physiological characteristics. In G. E. W. Wolstenholme & M. O'Connor (Eds.), *CIBA Foundation Colloquia on Ageing: the Lifespan of Animals* (pp. 1422–177). Churchill, London: J. & A.

Cheung, W. W. L., Dunne, J., Sarmiento, J. L., & Pauly, D. (2011). Integrating ecophysiology and plankton dynamics into projected maximum fisheries catch potential under climate change in the Northeast Atlantic. *ICES Journal of Marine Science*, *68*(6), 1008–1018. https://doi.org/10.1093/icesjms/fsr012

Cheung, W. W. L., Lam, V., & Pauly, D. (2008). *Modelling present and climate-shifted distribution of marine fishes and invertebrates.* (Fisheries Centre Research Reports No. 16(3)) (p. 72). University of British Columbia. Retrieved from http://hdl.handle.net/2429/40936

Hilborn, R., & Walters, C. J. (1992). *Quantitative Fisheries Stock Assessment*. Springer, Boston, MA.

Pauly, D., & Cheung, W. W. L. (2017). Sound physiological knowledge and principles in modeling shrinking of fishes under climate change. *Global Change Biology*, *24*(1), e15–e26. https://doi.org/10.1111/gcb.13831

Tai, T. C., Harley, C. D. G., & Cheung, W. W. L. (2018). Comparing model parameterizations of the biophysical impacts of ocean acidification to identify limitations and uncertainties. *Ecological Modelling*, *385*, 1–11. https://doi.org/10.1016/j.ecolmodel.2018.07.007
