# Supplementary material for: Potential socioeconomic impacts from ocean acidification and climate change effects on Atlantic Canadian fisheries
Source: PLoS One. 2020 Jan 10;15(1):e0226544. doi: 10.1371/journal.pone.0226544 (PMC6953801; doi:10.1371/journal.pone.0226544)
Supplement: S2 Note — (PDF) [file pone.0226544.s002.pdf]

Several fisheries in Atlantic Canada are distributed between “inshore” and “offshore” fisheries, with the main operational difference being vessel size. For most fisheries employment is concentrated in the inshore fishery, while a few larger vessels (with correspondingly larger crews) operate in the offshore. This was most relevant for the sea scallop (*Placopecten magellanicus*) fishery in NS. To account for this the NS data included estimates from two reports for offshore licence numbers and crew sizes (43, 47). The licence numbers were subtracted from the DFO reported number of licences (which do not differentiate between inshore and offshore) and calculated employment for both fisheries was summed.

Stimpsons’ surf clam (*Mactromeris polynyma*) licence data was collected from reports (rather DFO licences statistics) because DFO reported licence numbers represent all species of harvested clams, while the bulk of commercial surf clam harvest is concentrated under a very limited number of licences (51).
