# Supplementary material for: Potential socioeconomic impacts from ocean acidification and climate change effects on Atlantic Canadian fisheries
Source: PLoS One. 2020 Jan 10;15(1):e0226544. doi: 10.1371/journal.pone.0226544 (PMC6953801; doi:10.1371/journal.pone.0226544)
Supplement: S2 Table — (PDF) [file pone.0226544.s004.pdf]

S2 Table – Data sources for crew size estimates. Estimates from these sources were combined with number of licences (from DFO: http://www.dfo-mpo.gc.ca/stats/commercial/licences-permis/species-especes/se00-eng.htm) in the fisheries to arrive at an estimate of primary employment (except for shrimp, where primary employment numbers were derived directly from the industry report).

	LOBSTER	CRAB	SHRIMP	SCALLOP	CLAM
**NB – GULF**	DFO Costs and Earnings Survey (2004)	DFO Costs and Earnings Survey (2004)	Gardner-Pinfold consulting (shrimp - 2006)	*No data, used average crew size from other regions*	n/a
**NB – MAR**	DFO Costs and Earnings Survey (2004)	Gardner-Pinfold consulting (crab - 2006)	Gardner-Pinfold consulting (shrimp - 2006)	*No data, used average crew size from other regions*	n/a
**NEWFOUNDLAND + LABRADOR**	*No data, used average crew size from other regions*	DFO Costs and Earnings Survey (2004)	Gardner-Pinfold consulting (shrimp - 2006)	*No data, used average crew size from other regions*	n/a
**NS – GULF**	DFO Costs and Earnings Survey (2004)	DFO Costs and Earnings Survey (2004)	Gardner-Pinfold consulting (shrimp - 2006)	*No data, used average crew size from other regions*	n/a
**NS – MAR**	DFO Costs and Earnings Survey (2004)	Gardner-Pinfold consulting (crab - 2006)	Gardner-Pinfold consulting (shrimp - 2006)	DFO Costs and Earnings Survey (2004)	Michael Gardner – personal communication
**PEI**	DFO Costs and Earnings Survey (2004)	DFO Costs and Earnings Survey (2004)	Gardner-Pinfold consulting (shrimp - 2006)	*No data, used average crew size from other regions*	n/a
**QUE**	DFO Costs and Earnings Survey (2004)	DFO Costs and Earnings Survey (2004)	Gardner-Pinfold consulting (shrimp - 2006)	*No data, used average crew size from other regions*	*Crew size estimate from scallop fisheries, since fishery is very different from NS-Mar Surf Clam fishery*

DFO. (2007). Costs and earnings survey 2004: Atlantic region report (p. 72). Ottawa: Fisheries and Oceans Canada. Retrieved from http://publications.gc.ca/collections/collection_2012/mpo-dfo/Fs23-501-2004-eng.pdf

Gardner Pinfold Consulting. (2006). Profile of the Atlantic shrimp industry (p. 50). The Atlantic Council of Fisheries an Aquaculture Ministers.

Gardner Pinfold Consulting. (2006). Overview of the Atlantic snow crab industry (p. 66). The Atlantic Council of Fisheries an Aquaculture Ministers.
