# Supplementary figures and images for: Potential socioeconomic impacts from ocean acidification and climate change effects on Atlantic Canadian fisheries
Source: PLoS One. 2020 Jan 10;15(1):e0226544. doi: 10.1371/journal.pone.0226544 (PMC6953801; doi:10.1371/journal.pone.0226544)

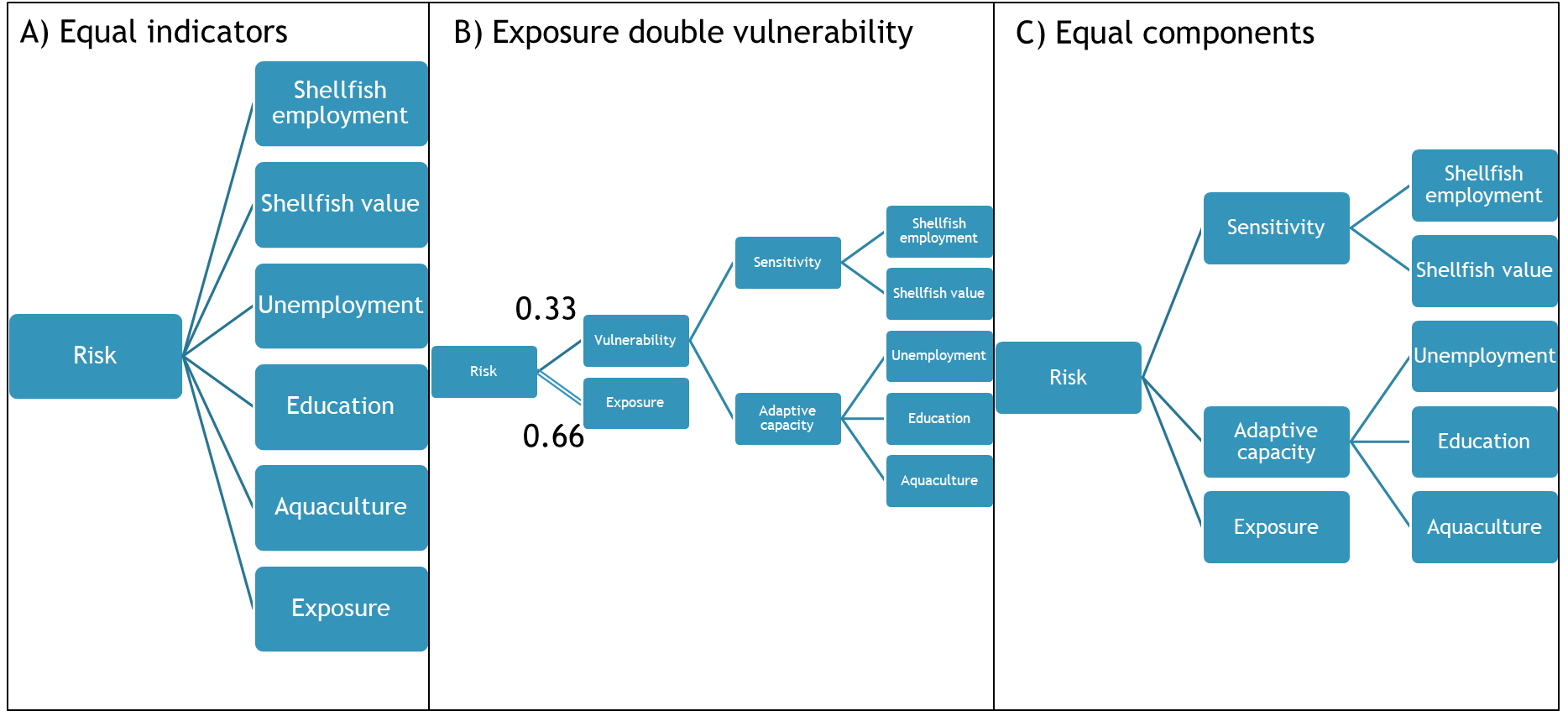

Supplement: S2 Fig — Letters correspond to sensitivity testing risk outcomes in S4 Table (TIF) [file pone.0226544.s007.tif]
